# Cell type-specific in vivo proteomes with a multicopy mutant methionyl tRNA synthetase mouse line

**DOI:** 10.1038/s41684-025-01589-2

**Published:** 2025-08-13

**Authors:** Rodrigo Alvarez-Pardo, Susanne tom Dieck, Kristina Desch, Belquis Nassim Assir, Cristina Olmedo Salinas, Riya S. Sivakumar, Julian D. Langer, Erin M. Schuman, Beatriz Alvarez-Castelao

**Affiliations:** 1 Biochemistry and Molecular Biology Department, Veterinary School, Complutense University of Madrid, Madrid, Spain; 2 Max Planck Institute for Brain Research, Frankfurt am Main, Germany; 3 San Carlos Clinical Hospital Health Research Institute, IdISSC, Madrid, Spain

**Keywords:** Protein-protein interaction networks, Neurotransmitters

## Abstract

The functional diversity of cells is driven by the different proteins they express. While improvements in protein labeling techniques have enabled the measurement of proteomes with increased sensitivity, measuring cell type-specific proteomes in vivo remains challenging. One of the most useful pipelines is bio-orthogonal noncanonical amino acid tagging (BONCAT) with the MetRS* system, consisting of a transgenic mouse line expressing a mutant methionyl-tRNA synthetase (MetRS*) controlled by Cre recombinase expression. This system allows cell type-specific labeling of proteins with a noncanonical amino acid (azidonorleucine, ANL), which can be subsequently conjugated to affinity or fluorescent tags using click chemistry. Click-modified proteins can then be visualized, purified and identified. The reduction in sample complexity enables the detection of small changes in protein composition. Here, we describe a multicopy MetRS* mouse line (3xMetRS* mouse line) that exhibits markedly enhanced ANL protein labeling, boosting the sensitivity and temporal resolution of the system and eliminating the need for working under methionine depletion conditions. Cell type-specific in vivo labeling is possible even in heterozygous animals, thus offering an enormous advantage for crossing the line into mutation and disease-specific backgrounds. Using the 3xMetRS* line, we identified the in vivo proteome of a sparse cell population—the dopaminergic neurons of the olfactory bulb—and furthermore determined newly synthesized proteins after short labeling durations following a single intraperitoneal ANL injection.

## Abstract (web summary)

The authors describe the second generation of the mutant methionyl tRNA synthetase mouse line, designed to increase mutant enzyme expression, enhance the labeling of nascent proteins and facilitate cell type-specific proteome identification.

## Main

Cellular protein composition and how it is modified in response to physiological and pathological signals and events is key to understanding how cells function. It is clear that the same extra- or intracellular signals can lead to distinct proteomic responses in different cell types^1,2^. Indeed, bulk analyses of proteomes can ‘average out’ intrinsic cellular differences as well as cell type-specific differences in responses. One way to obtain cell type-specific proteomes exploits cell type-specific markers and fluorescent labeling, followed by enrichment using fluorescence-activated cell sorting for subsequent mass spectrometry (MS) studies. A drawback of this approach, however, is the loss of processes and compartments, such as dendrites or axons, during sample processing^3,4^. By contrast, BioID- and TurboID-based methods are explicitly designed to identify proteins from targeted subcellular regions. Because protein populations are labeled in a proximity-dependent manner at a specific time point, this method includes spatial and temporal information but does not distinguish between newly synthesized and preexisting proteins^5^.

Bio-orthogonal methods based on the use of artificial amino acid analogs as baits for protein visualization and purification are particularly amenable to the study of cell type-specific proteomes^6,7^. The incorporation of artificial amino acids into proteins can be genetically controlled to enable cell type-specific labeling^8,9^. For instance, the expression of a mutant methionyl-tRNA synthetase with an enlarged methionine recognition pocket (L274G point mutation; hereafter referred to as MetRS*) allows the charging of the methionine analog azidonorleucine (ANL) to the corresponding methionine tRNA. ANL has a slightly larger structure compared with methionine. This size difference is sufficient to exclude ANL recognition by the endogenous MetRS; thus, ANL is not incorporated into proteins in cells where MetRS* is not present^10^. When MetRS* expression is driven by cell type-specific promoters, it enables specific labeling of proteomes with ANL and, after tissue lysis or fixation, derivatization of the azide group present in ANL with an alkyne by click chemistry. The alkyne can be used to visualize cell type-specific proteomes by immunofluorescence (FUNCAT) or western blot (BONCAT) and to purify these proteomes. The MetRS* system overcomes the above-mentioned challenges: it is possible to obtain cell type-specific proteomes without losing proteins located in dendrites or axons^11^. In addition, the time window of protein synthesis is determined by the exposure time of ANL, and protein degradation or subcellular location of ANL-labeled proteins is not altered; using this method, it is even possible to measure protein half-lives^8,12^.

Based on this system, we originally created a mouse model carrying a knock-in of the MetRS* gene fused to green fluorescent protein (GFP) by a P2A peptide under an enhanced actin promoter. Expression of the cassette is switched on by Cre-dependent excision of a transcriptional stop. Crossing this mouse line with cell type-specific Cre drivers enables cell type-specific protein labeling and its subsequent visualization, purification and identification. Given the considerable number of Cre drivers, the described system can be used to study cell type-specific proteins from almost any tissue in any field. Proteins can be labeled with ANL in live animals or in vitro. Using this first generation of mice, we demonstrated that it is possible to purify and identify cell type-specific proteomes allowing the identification of the respective cell type of origin. Furthermore, we identified proteins whose expression in the excitatory hippocampal neurons is modified in response to an enriched environment, a well-established paradigm for synaptic plasticity enhancement^8^. It is also possible to identify changes in protein expression in models of prion diseases^13^. However, we observed limitations when we aimed to obtain proteomes from relatively low-abundance cell types. Similarly, the identification of proteins labeled for short periods was not possible in brain tissue using this mouse model. Furthermore, given that the line expresses a single copy of the MetRS*, we probably observed substantial competition with methionine incorporation by the endogenous MetRS. Indeed, to obtain good in vivo labeling, a low-methionine diet and homozygous MetRS* alleles were required. The need for homozygosity complicates the crossing of this mouse line into a disease or any other background of interest. To overcome these limitations, we describe here a second generation of the MetRS* mouse tool designed to increase the expression of the mutant enzyme. The new design of the line boosts ANL incorporation into proteins, allowing the isolation and identification of low-number neuronal populations, such as the dopaminergic (DA) neurons, and proteins synthesized in the excitatory neurons of the cortex after just 3 h of a single intraperitoneal (IP) injection of ANL.

## Results

### The 3xMetRS* mouse line

**Fig. 1:**
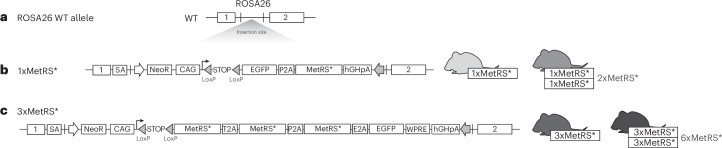
Comparison of the two mouse lines for ANL cell type-specific protein labeling. **a**, Insertion of the MetRS* cassette in the ROSA26 locus of wild-type mice (WT). **b**, Scheme of the cassette introduced in the first generation of the floxed-STOP-MetRS* mouse line. The right part shows mice expressing one (1xMetRS*, heterozygous animals) or two (2xMetRS*, homozygous animals) copies of the transgene after recombination by Cre. **c**, Scheme of the cassette introduced in the second-generation floxed MetRS*, showing all the introduced genes separated by 2A peptides, and the mRNA stabilization element (WPRE) at the end of the cassette. The right part shows mice expressing three (3xMetRS*, heterozygous animals) or six (6xMetRS*, homozygous animals) copies of the gene after recombination by Cre. SA, splice acceptor.

To achieve more efficient ANL labeling to purify proteomes from sparse cell populations or from proteomes after short ANL labeling times, we focused on improving the expression of MetRS*. We therefore modified and optimized the allele design of the previous MetRS* mouse line (termed 1xMetRS* here). We maintained the overall strategy of a Cre-dependent excision of a floxed transcriptional stop to switch on transcription of a cassette with MetRS* and GFP in the ROSA26 locus (Fig. 1a). Whereas the 1xMetRS* line carried a GFP sequence fused to the MetRS* coding region, separated by the self-cleaving 2A peptide sequence (P2A) (Fig. 1b), the new MetRS* mouse line described here (hereafter referred to as 3xMetRS*) expresses a cassette carrying three copies of MetRS* coding sequences and one copy of GFP per allele, with all four parts separated by sequences coding for different self-cleaving 2A peptides (Fig. 1c). As this expression cassette codes for a very large mRNA (and protein before separation of the single units by self-cleavage), we added an mRNA stabilization element (WPRE) to boost expression of the exogenous mRNA. In addition, we shifted the GFP to the C-terminus of the encoded protein (Fig. 1c). As such, we ensured that, if GFP is detected, MetRS* is also translated. Mice carrying the 3xMetRS* allele and the Cre::3xMetRS* lines used in this work were viable and fertile, and both heterozygous and homozygous mice showed no obvious behavioral differences compared with wild-type mice. We use the MetRS* copy numbers to refer to the genotype: 1xMetRS* and 2xMetRS* when studying heterozygous or homozygous animals, respectively, in the previously described mouse line; and 3xMetRS* or 6xMetRS* when using heterozygous or homozygous animals of the new line (Fig. 1).

### Neuronal ANL labeling in vitro with different MetRS* copy numbers

**Fig. 2:**
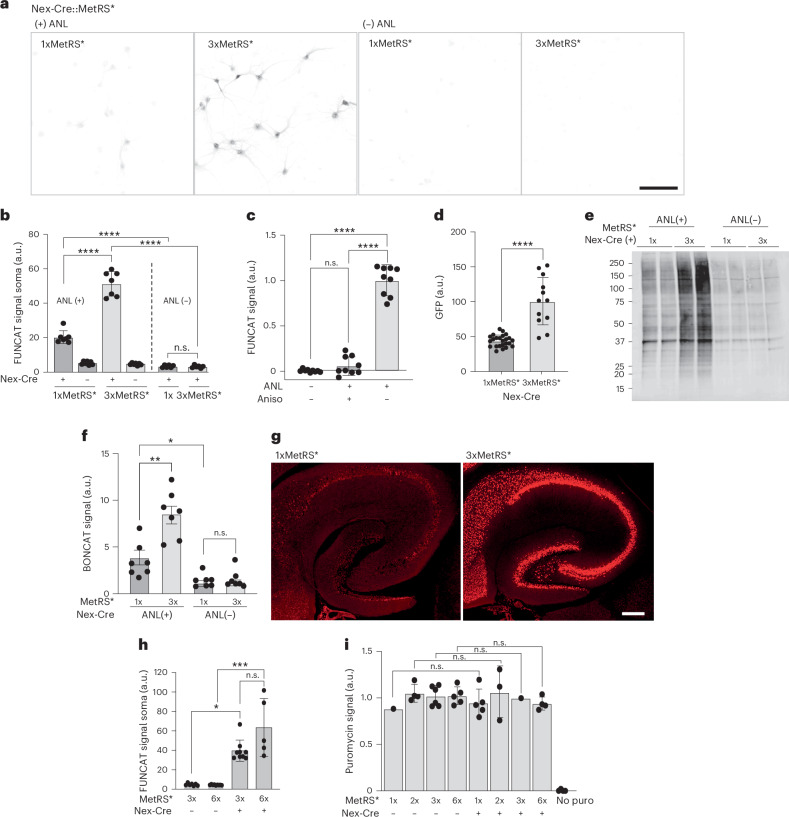
MetRS* copy number influences ANL labeling. **a**, Representative confocal images showing ANL labeling by FUNCAT after 2 h of incubation, in primary cortical neurons from Nex-Cre::1xMetRS* or Nex-Cre::3xMetRS* mice with (+) or without (−) ANL, showing an increase in labeling in the 3xMetRS* mouse line. Scale bar, 100 µm. **b**, Quantification of the experiments shown in **a**. Multiple-comparison ANOVA, ****P < 0.0001; data points shown are the mean of each dish with total cell numbers: 1x+: 453; 1x−: 1,153; 3x+: 1,034; 3x−: 676; 1x no ANL: 1,508; 3x no ANL: 1,304. **c**, Quantification of ANL incorporation in primary cortical neurons from Nex-Cre::3xMetRS* mice; ±anisomycin showing the specificity of the labeling for ANL incorporated into proteins. Multiple-comparison ANOVA, ****P < 0.0001. Full image quantification was performed from three independent experiments, each including three dishes per condition (total *n* = 9 dishes per condition). For each dish, 10 images were acquired and averaged, resulting in a total of 90 images analyzed per condition; each shown data point represents the mean of one dish. **d**, GFP expression in Nex-Cre::1xMetRS* and Nex-Cre::3xMetRS* cultured cells quantified by confocal microscopy. Multiple-comparison ANOVA, ****P < 0.0001; 3xMetRS* *n* = 12; 1xMetRS* *n* = 24 dishes (with total cell numbers 3x: 3,869; 1x: 1,372). **e**, A representative western blot showing BONCAT for ANL labeling in cortical primary neurons from Nex-Cre::1xMetRS* and Nex-Cre::3xMetRS* mice ± ANL. **f**, A graph displaying the quantification of the experiments shown in **e**. Unpaired *t*-test, *P = 0.0314, **P = 0.0011. **g**, Representative confocal images of FUNCAT in acute slices from Nex-Cre::1xMetRS* and Nex-Cre::3xMetRS* mice labeled with ANL for 2 h, showing newly synthesized proteins in the excitatory neurons of the hippocampus. Scale bar, 250 µm. **h**, Quantification of ANL incorporation in primary cortical neurons from Nex-Cre::3xMetRS* and Nex-Cre::6xMetRS* mice, showing increased ANL labeling compared with no-Cre control, with a nonsignificant increase between Nex-Cre::3xMetRS* and Nex-Cre::6xMetRS cells. Data points are mean per dish (number of dishes (cells) is 3x control: 7 (905); 6x control: 9 (1,422); 3x Cre: 9 (1,170); 6x Cre: 5 (703)). Multiple-comparison ANOVA, ***P = 0.0010, *P = 0.270**. i**, Quantification of puromycin incorporation in primary cortical neurons from heterozygous or homozygous 1xMetRS* and 3xMetRS* mice showing similar incorporation of puromycin ± Cre. Data points represent dishes (number of quantified neurons is 1x Cre: 979; 2x Cre: 428; 3x Cre: 286; 6x Cre: 286; 1x control: 171; 2x control: 951; 3x control: 690; 6x control: 337; 1x Cre no puro: 134; 2x Cre no puro: 108; 2x control no puro: 163; 6x control no puro: 231). Multiple-comparison ANOVA. The bars and error bars represent the mean ± s.d. in all panels. a.u., arbitrary units; n.s., not significant. Source data

To analyze the utility of increasing the MetRS* copy number, we compared ANL incorporation between the original and second-generation lines. First, we evaluated whether there was increased ANL labeling in the 3xMetRS* line compared with the 1xMetRS* line in vitro. We crossed animals carrying the respective alleles to the Nex-Cre driver line, driving Cre expression to excitatory neurons^14^, and performed experiments on cultured cortical neurons derived from the offspring. Experiments in culture have the advantage that ANL labeling is direct and circumvents any variability arising from ANL administration or uptake into the brain. Comparison of 2-h ANL labeling in either 1xMetRS* or 3xMetRS* excitatory neurons (Nex-Cre::1xMetRS* and Nex-Cre::3xMetRS*) evaluated by FUNCAT showed that ANL labeling was approximately three times higher in neurons expressing 3xMetRS* (Fig. 2a,b)^12^. FUNCAT experiments in the presence of the protein synthesis inhibitor anisomycin (Fig. 2c) confirmed that the observed fluorescent signal was due to ANL incorporation into newly synthesized proteins and not due to detection of free ANL or ANL-charged tRNAs. Interestingly, we observed that the increased ANL labeling in the Nex-Cre::3xMetRS* line compared with Nex-Cre::1xMetRS* was accompanied by higher GFP expression—even though both lines carry one copy of GFP (Fig. 2d). Similarly, western blot comparisons of ANL labeling in 1xMetRS* or 3xMetRS* cultured neurons by BONCAT also showed an increase in ANL labeling (Fig. 2e,f). To examine whether the observed increase in labeling with the 3xMetRS* line was also evident in tissue, we prepared acute slices from brain tissue of adult Nex-Cre::1xMetRS* or 3xMetRS* mice and performed in vitro slice metabolic ANL (2 h) labeling experiments. As was observed in the cultured neurons, FUNCAT labeling was substantially elevated in slices expressing the 3xMetRS* transgene (Fig. 2g).

To examine how the number of allele copies influences ANL labeling, we prepared primary cortical cultures from offspring of crosses leading to different genotypes and compared Nex-Cre::3xMetRS* and Nex-Cre::6xMetRS* with their littermates that did not express Cre. We observed only a slight increase in ANL labeling in 6xMetRS* versus 3xMetRS* in cultured neurons (Fig. 2h), suggesting that the maximal labeling may plateau under our conditions. In contrast to the copy number dependence of the ANL labeling, we found, as expected, that total protein synthesis levels, as assessed by puromycylation^15^, were not different between heterozygous or homozygous 1xMetRS* and 3xMetRS* (Fig. 2i). Thus, ANL incorporation depends, as expected, on Cre expression and the rank order of ANL incorporation scales depending on MetRS* expression. The scaling between ANL incorporation and encoded MetRS* copy number, however, was not linear and could be influenced by additional factors. For example, there could be a difference in the mRNA abundance of the transcripts transcribed from the 1xMetRS* and 3xMetRS* alleles. As the large expression cassette in the 3xMetRS* line (3.6 kb for 1xMetRS* versus 8.6 kb for 3xMetRS*) was expected to be transcribed and translated poorly, we added a WPRE stabilization element to this construct to ensure expression. As a result, the 3xMetRS* cassette expression may be higher. To understand whether mRNA abundance contributes to the superior performance of the 3xMetRS* line and the difference in GFP protein expression, we conducted fluorescence in situ hybridization (FISH) experiments using probes to detect *gfp* mRNA, cell-type marker mRNAs (*Gad2*, *Camk2a* and *Bdnf*) and the MetRS mRNA *Mars1*^16^. As only excitatory neurons are expected to express the mutant MetRS* in the crosses with Nex-Cre, we reasoned that inhibitory neurons (*Gad2*+) could serve as controls in these cultures (Supplementary Fig. 1a,c–e). Therefore, these assays allowed us to also assess the level of MetRS* transcript expression in relation to the wild-type transcript. Consistent with the observed higher GFP protein expression, mRNA expression of the inserted cassette in cultures was higher in the 3xMetRS* mouse compared with the 1xMetRS*, as judged by *gfp* FISH signal (Supplementary Fig. 1b) suggesting stabilization of the large transcript.

The *Mars1* FISH signal also revealed a substantial overexpression of the mRNA for MetRS* in the 3xMetRS* compared with the 1xMetRS* mouse (Supplementary Fig. 1b,d,e). Although the *Mars1* FISH probe does not discriminate between the mutant and wild-type sequence, we addressed the relative expression levels of mRNA for wild-type MetRS and mutant MetRS* by performing dual-color FISH labeling in various genotypes comparing distinct cell types (*gfp*+, *Gad2*+, *Camk2a*+ and *Bdnf*+) within the cultures from Cre-positive and Cre-negative animals (Supplementary Fig. 1c–e). In the absence of Cre, *Mars1* levels were comparable between excitatory and inhibitory cells, reflecting the levels of the wild-type enzyme mRNA. In the Nex-Cre::3xMetRS* cultures the signal was significantly higher in excitatory cells (*Camk2a*+ and *Bdnf*+) than in inhibitory cells (*Gad2*+), as expected (Supplementary Fig. 1c,d). In the Nex-Cre::1xMetRS* cultures, overexpression of *mars1* in excitatory neurons was substantially lower and not significantly higher than the wild-type enzyme mRNA (Supplementary Fig. 1b,e). We obtained similar results from FISH experiments on brain slices, where we analyzed the hippocampal CA1 region (Supplementary Fig. 1f–h). Taken together, the in vitro experiments demonstrated a significantly increased Cre-dependent labeling efficiency of the Cre-defined cell type in the 3xMetRS* line compared with the 1xMetRS*.

### Methionine competition in 3xMetRS* versus 1xMetRS* in vitro

**Fig. 3:**
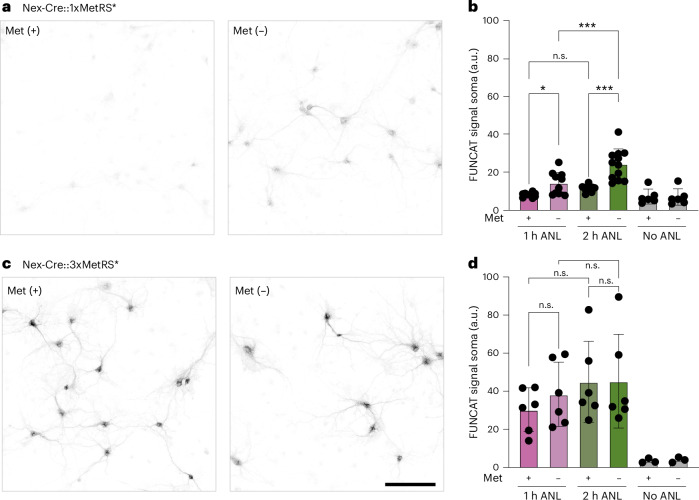
Methionine competition with ANL. **a**,**c**, FUNCAT in primary neurons from cortices of Nex-Cre::1xMetRS* mice (**a**) or Nex-Cre::3xMetRS* mice (**c**) labeled with ANL for 2 h in the presence (met (+)) or absence (met (−)) of methionine in the culture media. Scale bar, 100 µM. **b**,**d**, Quantification of the experiments shown in **a** (**b**) and **c** (**d**), including a 1-h time point and a no-ANL control. Multiple-comparison ANOVA was used to assess statistical significance, *P = 0.0437, ***P = 0.0001. Each dot represents the average of single-cell quantifications for one dish in at least three independent experiments with a minimum of two dishes per experiment. Number of neurons quantified are Nex-Cre::1xMetRS*, 1 h +met (2,085), 1 h −met (1,496), 2 h +met (1,904), 2 h −met (1,651); no ANL +met (1,189), no ANL −met (1,236); Nex-Cre::3xMetRS*, 1 h +met (814), 1 h −met (637), 2 h +met (558), 2 h −met (595), no ANL +met (520), no ANL −met (460). The bars and error bars represent the mean ± s.d. in all panels.

We reasoned that increasing the expression of MetRS* would favor the incorporation of ANL over methionine. To address this question, we compared ANL incorporation in Nex-Cre::1xMetRS* and Nex-Cre::3xMetRS* neurons in culture media supplemented with methionine or not. Indeed, ANL incorporation in Nex-Cre::3xMetRS* neurons was not only more pronounced than in Nex-Cre::1xMetRS* cultures; it was also not significantly inhibited by the presence of methionine in the growth medium, as observed in Nex-Cre::1xMetRS* neurons (Fig. 3 and Supplementary Fig. 2). While a slightly reduced (but nonsignificant) ANL uptake was still visible after 1 h of ANL incubation in Nex-Cre::3xMetRS* neurons in the presence of methionine, no difference was observed with 2-h labeling (Fig. 3c,d). This trend probably reflects the initial presence of methionine-loaded tRNA before the start of incubation. By contrast, Nex-Cre::1xMetRS* neurons exhibited strong methionine competition at both time points (Fig. 3a,b).

### ANL in vivo labeling in 2xMetRS* and 3xMetRS* mouse line

**Fig. 4:**
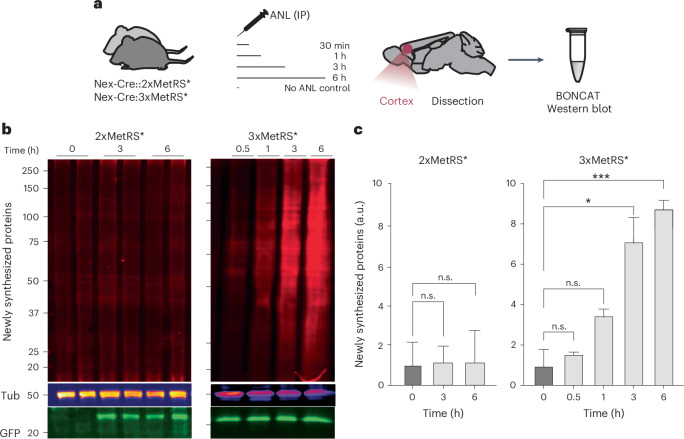
In vivo ANL labeling efficiency in 2xMetRS* versus 3xMetRS*. **a**, Experimental outline; Nex-Cre::2xMetRS* and Nex-Cre::3xMetRS* cortices were collected 0.5 h, 1 h, 3 h or 6 h after a single ANL IP injection. **b**, Western blot of newly synthesized proteins labeled with ANL and detected by BONCAT. Tubulin (Tub) is used as a control for protein loading, and GFP expression serves as a proxy for MetRS* expression. **c**, Quantification of the newly synthesized proteins shown in **b**. Multiple-comparison ANOVA, *P = 0.016 (0 h versus 3 h), ***P = 0.00051 (0 h versus 6 h) (*n* = 2 animals per condition done in two independent experiments). The bars and error bars represent the mean ± s.d. in all panels. Source data

The above enhanced in vitro labeling in Nex-Cre::3xMetRS* prompted us to examine in vivo ANL labeling. We administered ANL via a single IP injection and compared labeling from 0.5 to 6 h in the ‘best’ first-generation mouse line (homozygous, Nex-Cre::2xMetRS*) and the heterozygous mice from the new generation line (Nex-Cre::3xMetRS*) (Fig. 4a). As expected from earlier studies, no BONCAT signal was detectable after short labeling periods in cortex samples from the Nex-Cre::2xMetRS* mice; by contrast, protein synthesis was clearly detected from Nex-Cre::3xMetRS* mice with labeling times of 1 h or longer (Fig. 4b,c). This finding demonstrates that ANL incorporation is achieved much faster in the new mouse model, presumably due to enhanced efficiency of ANL loading and reduced methionine competition (Fig. 3 and Supplementary Fig. 2).

### ANL in vivo labeling in the 3xMetRS* and 6xMetRS* mouse line

**Fig. 5:**
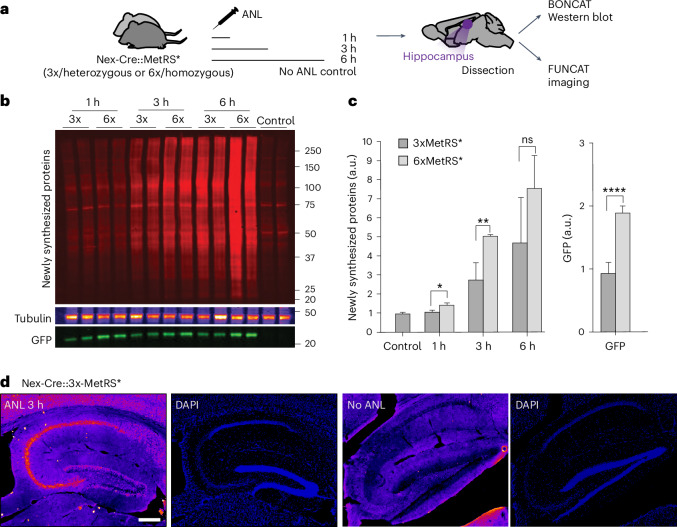
In vivo ANL labeling efficiency in 3xMetRS* versus 6xMetRS*. **a**, Experimental outline: ANL was administered to Nex-Cre::3xMetRS* and Nex-Cre::6xMetRS* mice via a single IP injection, and hippocampal tissue was collected after 1 h, 3 h and 6 h; non-ANL-injected mice were used as controls. **b**, Western blot of newly synthesized proteins labeled with ANL and detected by BONCAT. Tubulin was used as a loading control, and GFP serves as a proxy for MetRS* expression. **c**, Quantification of newly synthesized proteins and GFP expression. Unpaired Student’s *t*-test comparisons, *P = 0.038, **P = 0.0030, ****P < 0.0001. Left graph: *n* = 4 mice per group; right graph: *n* = 12 animals per group. **d**, FUNCAT images of Nex-Cre::3xMetRS* (heterozygous animals) and negative control brain sections collected 3 h after IP injection, showing specific ANL labeling in the excitatory neurons of the hippocampus. Scale bar, 200 µm. The bars and error bars represent the mean ± s.d. in all panels. Source data

To study the effect of allele copy number in the new line, we next compared heterozygous (Nex-Cre::3xMetRS*) and homozygous mice (Nex-Cre::6xMetRS*). As before, we analyzed the BONCAT signal from tissue labeled with ANL for short periods after a single IP injection to compare protein labeling during the exponential phase. Hippocampal tissue was collected at 1 h, 3 h and 6 h (Fig. 5a) after a single ANL injection, and protein synthesis was detected with increasing levels in both heterozygous and homozygous animals (Fig. 5b,c). While GFP expression was, as expected, doubled in the homozygous animals compared with the heterozygous ones, the BONCAT signal in the homozygous animals was higher as a trend at each of the studied time points, but slightly less than double (Fig. 5b,c). This finding fits with the findings from the in vitro experiments and suggests an initial saturation of tRNA loading. Thus, excitingly, even though labeling is slightly better in homozygous mice, it is still possible to use heterozygous animals for labeling. The protein synthesis signal is also detectable by FUNCAT in hippocampal sections from heterozygous animals after 3 h of labeling (Fig. 5d). We want to highlight that the ability to use heterozygous animals represents a substantial reduction in the breeding burden of mice, especially for experiments addressing cell type-specific proteomes in disease or mutation conditions.

### Identification of newly synthesized proteins via MS with temporal control

**Fig. 6:**
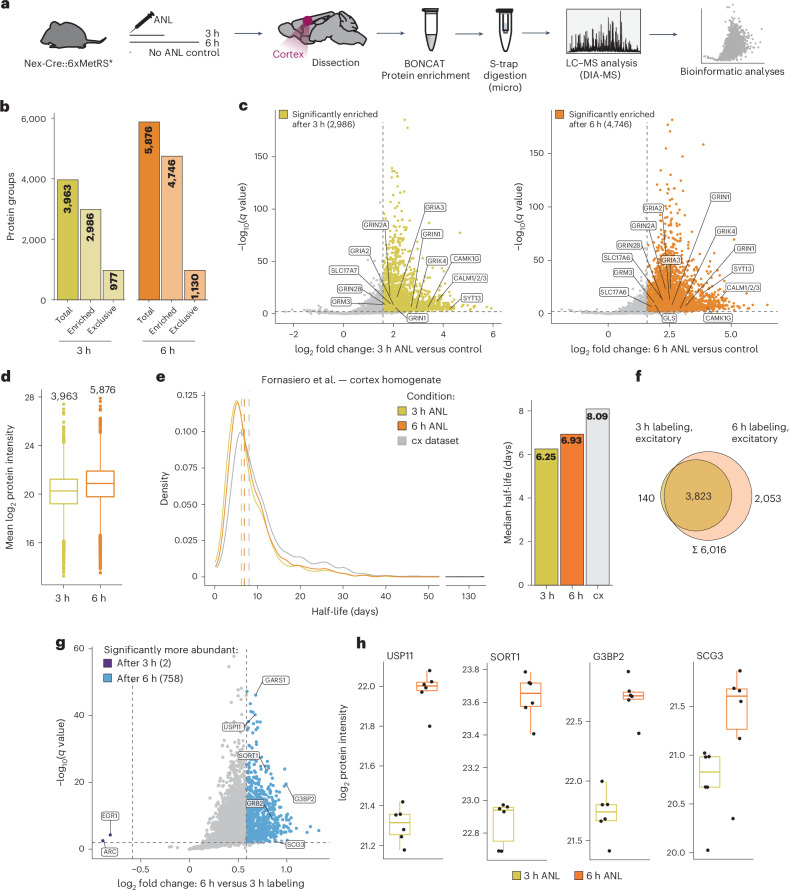
Excitatory neuronal protein identification after 3 h and 6 h of in vivo ANL labeling in Nex-Cre::6xMetRS*. **a**, The workflow for labeling and purification of excitatory neuronal proteins. Cortex tissue was collected 3 or 6 h after injection of ANL (*n* = 6 mice for each time point) or from no-ANL control (*n* = 2). Proteins containing ANL were clicked, purified and quantified by LC–MS. **b**, A bar plot showing protein group quantifications of the excitatory neuronal proteome after 3 and 6 h of labeling. Excitatory proteomes were defined by either exclusive identification in ANL-labeled samples or by significant enrichment over the unlabeled controls. See also Supplementary Fig. 3a. **c**, Volcano plots comparing the protein abundance of shared proteins in the 3- and 6-h samples. Significantly enriched protein groups are highlighted in color (3 h, yellow; 6 h, orange). **d**, A box plot of the newly synthesized excitatory proteins after 3 and 6 h of labeling. Significant increases in protein intensity were observed with prolonged labeling duration (Welch two-sample unpaired *t*-test, *P* < 0.0001). *n* numbers shown above each box plot. **e**, The density plot (left) shows the protein half-life distribution. Excitatory neuronal proteins identified were matched to a database for protein half-lives in the brain cortex^20^ and are highlighted by color; full cortex (cx) data are depicted in gray. Vertical lines highlight the increase in median half-lives of the matched excitatory proteins after 3 and 6 h (6.25 and 6.93 days) of labeling compared with all cortical proteins (8.09 days), which is summarized in the bar plot (right) as well. **f**, A Venn diagram comparing the excitatory proteomes of the two time points, indicating high overlap between them. **g**, A volcano plot comparing the shared excitatory protein groups quantified after 3 and 6 h of labeling. Significantly more abundant proteins (≥1.5-fold enrichment, FDR <0.01) are highlighted (3 h, purple; 6 h, blue). **h**, Box plots displaying selected candidate proteins showing significantly differential abundance between the 3- and 6-h time points. The box plots in **d** and **h** indicate the median and first and third quartiles, while whiskers extend to 1.5× interquartile range. DIA, data-independent acquisition.

To demonstrate the feasibility of identifying newly synthesized proteins using the 3xMetRS* mouse line, we identified nascent proteins from cortical excitatory neurons labeled for 3 h and 6 h (Fig. 6a, Supplementary Fig. 3a and Supplementary Data). We analyzed the proteins detected exclusively or enriched, with at least a threefold increase (false discovery rate (FDR) <0.01) compared with no-ANL control mice. We quantified more than 8,000 proteins in each dataset, of which 3,963 and 5,876 proteins were enriched or exclusive at 3 h and 6 h of labeling, respectively (Fig. 6b,c, Supplementary Fig. 3b–e and Supplementary Data). In addition, at 6 h, the overall intensity of the identified proteins significantly increased compared with the 3-h labeling time point (Fig. 6d). To confirm whether we could detect different protein pools by simply changing the ANL labeling period, and considering that newly synthesized proteins typically have shorter half-lives and are more dynamic^17,18^, we compared the obtained 3-h and 6-h proteomes with previously published protein half-life datasets in a density plot^19,20^ (Fig. 6e and Supplementary Fig. 3f). We found that the identified proteins at 3 h were shifted toward shorter (6.25 days, median) half-lives, while those at 6 h were shifted toward longer (6.95 days, median) half-lives, whereas the overall median half-life of the cortical proteins used as a background was 8.09 days (Fig. 6e). As such, by labeling with ANL for different durations, we alter the sensitivity for detecting proteins with different half-lives. Next, we compared the identified proteomes with each other (Fig. 6f–h and Supplementary Fig. 3g) and found 3,823 common proteins between both labeling times, with 140 and 2,053 proteins identified exclusively at 3 h and 6 h, respectively. Looking into the common proteins in both datasets, we found 758 proteins significantly more abundant at 6 h, while only 2 proteins were found to have higher intensities at 3 h (at least 1.5-fold, FDR <0.01).

### Identification of a cell type-specific proteome from a low-abundance neuronal population

**Fig. 7:**
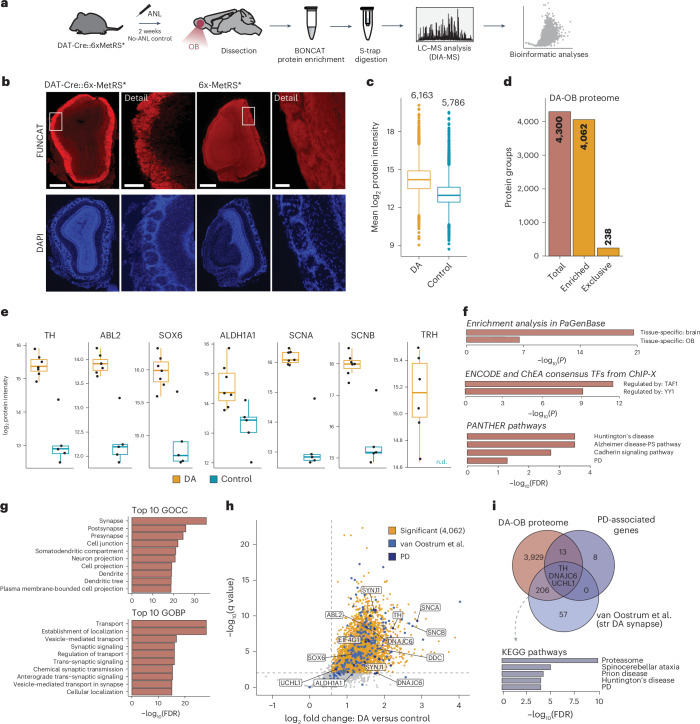
OB-DA proteome identification in DAT-Cre::6xMetRS*. **a**, The workflow for labeling and purification of OB-DA proteins. In vivo protein labeling was performed by daily IP injection of ANL for 2 weeks in DAT-Cre::6xMetRS* mice (ANL, *n* = 7; no-ANL control, *n* = 5). OB tissue was collected, and ANL-containing proteins were clicked, purified and quantified by LC–MS. **b**, Confocal microscopy images showing the ANL-labeled proteins by FUNCAT in the OB-DA neurons from DAT-Cre::6xMetRS* mice. Scale bars, 200 μM, 40 μM (DAT-Cre::6x-MetRS* detail) and 30 μM (6x-MetRS* detail). **c**, A box plot showing that most proteins of the OB-DA neuron samples are enriched over the control samples. *n* numbers shown above each box plot. **d**, A bar plot showing protein group identifications of the quantified OB-DA proteins. The OB-DA neuron proteome was defined by either exclusive identification or enrichment in ANL-labeled samples over the unlabeled controls. See also Supplementary Fig. 5a. **e**, Box plots displaying brain disease-associated and/or previously reported DA proteins identified in the DA-OB proteome. n.d., not found. **f**, Overrepresentation analysis showing enriched terms for the DA proteome according to PaGenBase, transcription factor (TF) ChIP-seq data from ENCODE and cellular pathways by PANTHER. **g**, Gene Ontology overrepresentation displaying the top ten terms for the DA proteome, based on gene ontology cellular compartment (GOCC) and biological process (GOBP) annotations (FDR <0.05). **h**, A volcano plot comparing proteins quantified in labeled samples with the unlabeled controls. Significantly enriched protein groups (80% valid value in labeled condition, at least 1.5-fold enrichment, FDR <0.01) are highlighted in yellow. Furthermore, color indicates proteins matching the DA proteome of striatal synapses or PD-associated genes (UniProt KW: KW-0907). **i**, A Venn diagram highlighting the overlap of the OB-DA proteome with DA proteins of striatal (str) synapses or PD-associated genes (UniProt KW: KW-0907). Genes shared between the OB-DA neuron proteome and the striatal DA synapse proteome from van Oostrum et al.^30^ show an enrichment in proteasome and neurodegenerative disease proteins (Kyoto Encyclopedia of Genes and Genomes (KEGG) pathways, FDR <0.05). Box plots in **c** and **e** specify the median and first and third quartiles, and whiskers extend to 1.5× interquartile range. Analyses in **f** and **g** were done using OB background.

Given the increased labeling sensitivity in the 3xMetRS* mouse line, we next tested whether we could identify proteomes from neurons that account for low numbers in a given brain region. As a proof of principle, we chose the DA neurons from the substantia nigra (SN) and the olfactory bulb (OB). In the SN, there are approximately 14,000 DA neurons in each mouse^21^; and in the OB, the DA neurons represent 6% of the OB cells, totaling about 89,000 cells^22^. By pooling dissected SN from several animals (five per replicate), we were able to detect nascent protein labeling by BONCAT. However, the amount of purified protein was not sufficient for accurate protein identification following MS. Thus, we next focused on the OB and identified, for the first time, the DA neuron-OB proteome, using DAT-Cre::6xMetRS* animals labeled with ANL for 2 weeks (Fig. 7a,b, Supplementary Figs. 4 and 5a and Supplementary Data).

We identified 4,300 proteins derived from the DA neurons of the OB (Fig. 7c,d, and Supplementary Fig. 5b–e), of which 4,062 were enriched by at least 1.5-fold in the OB-DA neuron dataset (compared with no-ANL control mice) and 238 were exclusive to the labeled conditions (Fig. 7d,e). We used several databases, including Metascape^23,24^, to confirm that the identified proteins in our OB-DA neurons are specific to the OB and brain tissue. Overrepresentation analysis of the OB-DA proteome compared with a full OB proteome (used as background^25^) in the Enrichr database^26–28^ with transcription factor chromatin immunoprecipitation sequencing (ChIP-seq) data from ENCODE indicated increased expression of genes regulated by TAF1 and YY1 (Ying Yang 1) (Fig. 7f). We also performed an overrepresentation analysis (using the PANTHER pathway database^29^) and found terms associated with three neurodegenerative diseases: Alzheimer’s disease, Huntington’s disease and Parkinson’s disease (PD) (Fig. 7f). Furthermore, as expected, there was pronounced overrepresentation of Gene Ontology terms related to neurons and synapses (Fig. 7g). Next, we compared the DA-OB proteome with the proteome of DA neuronal synaptosomes isolated from the striatum and detected several common proteins (209)^30^. These common proteins show significant enrichment in terms related to the proteasome, which has an important role in PD and other proteinopathies (Fig. 7h,i).

## Discussion

Understanding how protein homeostasis works in vivo is essential for fully grasping the internal processes of cells and organisms. Complex organisms comprise hundreds of distinct cell types, each responding differently to identical physiological or pathological challenges owing to their specialization. The method described here uses the expression of a mutant MetRS*, which allows the incorporation of a bio-orthogonal analog of methionine (ANL) into proteins, enabling protein purification and increasing detection sensitivity by MS. In the present work, we compared a first-generation mouse line expressing one copy of the MetRS* enzyme per allele (1xMetRS*) with a second-generation line expressing three copies of MetRS* (3xMetRS*) per allele. In both mouse lines, MetRS* expression is Cre recombinase dependent. With the 3xMetRS* line, we increased the ANL labeling efficiency, reduced the time of labeling to study protein synthesis over a shorter time frame, and identified a cellular proteome in a very small population of DA neurons.

One drawback of metabolic labeling with amino acid analogs is competition with the corresponding natural amino acid. To achieve effective labeling, experiments with the first-generation MetRS* line—both in vitro and in vivo—were necessarily conducted under methionine-free or methionine-reduced conditions to prevent competition between methionine and ANL. Here, we obtained strong labeling without altering physiological methionine concentrations. We demonstrated that cell type-specific protein identification is robust just 3 h after a single ANL IP injection. In addition, when comparing the proteins identified after 3 h with those identified after 6 hours, we identified protein pools with different half-lives, highlighting the potential of the 3xMetRS* system to study newly synthesized proteins that are missed with the 1xMetRS* system.

The growing interest in the study of proteins and their behavior has led to the continuous development of methods and various techniques^8,15,31–36^. Despite this progress, BONCAT^6^, particularly ANL labeling, remains the most versatile method for studying protein homeostasis in vivo. The strength of this system lies in its cell type specificity, allowing in situ visualization of proteins, and its reliance on metabolic labeling. Depending on the labeling duration, ANL can be utilized to investigate different aspects of protein homeostasis: (1) studying protein synthesis by identifying proteins after brief labeling periods; (2) examining protein steady state or proteomes by identifying proteins after longer labeling periods that exceed the average half-lives of the proteins in the studied tissue or cell type; and (3) analyzing protein degradation by ceasing ANL administration after an initial labeling (*t* = 0) and tracking the decrease of labeled proteins over time. Moreover, the possibility to identify the cell type-specific proteomes from one single animal opens the door to grouping mice on the basis of their behavior, for instance, opening a new dimension for functional studies. Other methods, such as puromycylation, are extensively used for studying protein synthesis owing to their high sensitivity. Puromycin is incorporated at any location within the translating protein. This method allows the investigation of protein synthesis after short labeling periods (for example, 5–10 min). However, prolonged labeling with puromycin can lead to cellular toxicity, and the method is not cell type specific. However, recent developments have enabled puromycin resistance or activation in identified cell types^36,37^. Another useful approach is TurboID and its variations. This method relies on biotin binding to proteins in close proximity through an enzymatic reaction. Like puromycylation, BioID is highly efficient, allowing brief labeling periods. However, it is primarily used to study local proteomes or protein–protein interactions and is not suitable for studying protein synthesis, as it does not involve metabolic labeling^38,39^. Meanwhile, the BONCAT system is being expanded to include additional noncanonical amino acids (ncAAs) such as AzY and AzF^40^, although so far no systematic comparison of their catalytic efficiency relative to MetRS*/ANL has been performed. BONCAT efficiency depends on many biological factors, including ncAA uptake, enzyme expression, codon usage and translational activity. The choice of the best BONCAT variant will depend on tissue, cell type, the exact experimental purpose and timing. For instance, the hydrophobicity of Phe and Tyr may reduce the accessibility of their ncAA analogs in folded proteins for applications such as live-cell labeling or FUNCAT, making ANL a more suitable choice for these applications; however, this might not be as important for BONCAT, which is performed under denaturing conditions. The combination of different BONCAT variants, either simultaneously or sequentially (for example, with the development of alkyne analogs of the ncAAs to allow pulse-chase experiments), will enrich the toolbox for studying protein dynamics and sensitivity.

The 3xMetRS* line also enables the identification of proteomes from low-abundance neuronal populations, such as OB-DA neurons (representing just 6% of the cells in the bulb). Regrettably, we were for now unable to purify and identify the proteome from the DA neurons of the SN using a similar number of neurons as starting material. This may indicate that the protein synthesis rate of DA neurons in the bulb is higher, probably influenced by the retained capacity of the OB-DA neurons for postnatal proliferation. Indeed, this feature positions the OB-DA neurons as potential instruments for restorative treatments for PD.

## Methods

### Transgenic animals

For the new mouse line, the cassette—STOPFLOX-3x(MetRS*-2A)-GFP—is expressed under the CAG actin-derived promoter. The line was custom made for us by Taconic GmbH by knock-in at the mouse ROSA26 locus using recombination-mediated cassette exchange in embryonic stem cells. For expression of the cassette in excitatory neurons, homozygous floxed-Stop 3xMetRS* (available upon request to B.A.-C., until available by JAX #040661; C57BL/6-*Gt(ROSA)26Sor*^tm2(CAG-GFP,-Mars*L274G)Esm^/J) animals or MetRS* animals (JAX #028071; C57BL/6-*Gt(ROSA)26Sor*^tm1(CAG-GFP,-Mars*L274G)Esm^/J) were crossed with homozygous Nex-Cre mice (kindly provided by K. A. Nave)^14^, or DAT-Cre mice (JAX #006660)^41^. Genotyping of the 3xMetRS* and wild-type alleles was conducted by PCR in separate reactions with KAPA2G Fast (HotStart) Genotyping Mix (WT allele: primers CTC TTC CCT CGT GAT CTG CAA CTC C, CAT GTC TTT AAT CTA CCT CGA TGG; internal control primers: GAG ACT CTG GCT ACT CAT CC, CCT TCA GCA AGA GCT GGG GAC; knock-in allele primers: ACT GGC TAC CTT GTC TGA GGA G, CAT GGA TGG CAT GGT ACT TG, 94 °C 1 min, 35× (94 °C 30 s, 62 °C 30 s, 72 °C 1 min) 72 °C 10 min, 4 °C). All experiments involving animals were performed with permission from the local government offices in Germany (RP Darmstadt; protocol V54-19c20/15-F126/2002) or Spain (Committee of Animal Experiments at UCM and Environmental Counseling of the Comunidad de Madrid, protocol number PROEX 005.0/21). These experiments comply with the German Animal Welfare Law, Max Planck Society rules and Spanish regulations and follow European Union (EU) guidelines for animal welfare. Mice were housed in accordance with EU Directive 2010/63/EU Annex III and euthanized using methods in accordance with EU Directive 2010/63/EU Annex IV.

For in vitro experiments, tissue for cultures or slices was extracted from F1 offspring of crosses from females homozygous for Nex-Cre and males homozygous for 1x/3xMetRS* or from crosses of F1 females (heterozygous for both genes) to homozygous 1x/3xMetRS* males. For in vivo experiments, F1 mice (heterozygous for both genes) were bred together. Animals with two copies of 3xMetRS* were again bred together until obtaining animals homozygous for both genes (3xMetRS and Cre), and the colony was maintained in this way for reasons of animal number reduction. This maintenance scheme can be carried out only for lines with low incidence of germline activation. We regularly check for off-target expression and/or germline activation in the lines by FUNCAT and/or GFP staining. Of note, for Cre driver lines, whether Cre is transmitted maternally or paternally may be important, depending on specific recommendations. In addition, we assessed recombination-mediated loss of target gene by looking for processing intermediates of 2A self-cleavage products and found no evidence for copy loss.

### Antibodies

The following antibodies were used for immunofluorescence (IF) labeling and/or immunoblotting (IB) at the indicated dilutions. Primary antibodies: rabbit anti-biotin (IB, 1:1,000, Cell Signaling 5597), rabbit anti-GFP (IF in cells 1:500, abcam ab6556) or chicken anti-GFP (IF, 1:500; IB: 1:1,000; Aves), chicken anti-GFP (IB, IF 1:500, abcam ab13970), guinea pig anti-MAP2 (IF in cells 1:1,000, Synaptic Systems 188004) and mouse anti-puromycin (IF in cells 1:3,500, Kerafast EQ0001). Secondary antibodies: goat anti-mouse, anti-rabbit or anti-chicken IRdye680 or IRdye800 (IB, 1:10,000, Licor), goat anti-guinea pig-Dylight405 (IF in cells 1:1,000, Jackson ImmunoResearch 106-475-003), goat anti-rabbit Alexa488 (IF in cells, 1:1,000, Thermo Fisher Scientific A11008), goat anti-chicken Alexa488 (IF in slices, 1:1,000, Thermo Fisher Scientific A11099) and goat anti-mouse Alexa546 (IF in cells 1:1,000, Thermo Fisher Scientific A11030). In slice IF experiments, 4′,6-diamidino-2-phenylindole (DAPI) was added to the secondary antibody solutions or wash solutions where appropriate.

### Primary cultures

Primary neuronal cultures were prepared from pooled litters or single animals of either sex from crosses of homozygous Nex-Cre females with homozygous 1x/3x MetRS* males or double-heterozygous Nex-Cre::1x/3xMetRS* females with the respective homozygous 1x/3xMetRS* males and maintained as previously described^42^. In brief, cortices from newborn postnatal 0/1 (P0/P1) transgenic mice, were dissected and dissociated with papain (Sigma P3125), washed, triturated and plated at a density of 30,000 cells per coverslip on poly-d-lysine-coated MatTek glass-bottom dishes (MatTek P35G-1.5-14C) for imaging experiments or 3 million cells on poly-d-lysine-coated 60-mm cell culture dishes for BONCAT. Neurons were maintained at 37 °C and 5% CO_2_ in NGM (Neurobasal-A plus B27 and GlutaMAX) supplemented with glia- and cortex-conditioned NGM. Experiments were performed after 7–14 days in vitro.

### BONCAT in cultured neurons

Growth medium was removed and replaced with NGM without methionine (Gibco, custom preparation) supplemented with 4 mM ANL (Iris Biotech HAA1625) or 4 mM methionine in the ANL(−) control. Following metabolic labeling for 2 h, cultured neurons were washed two times with D-PBS (Gibco) on ice and collected in D-PBS by scraping and a 40-s spin-down in a small bench top mini centrifuge. The supernatant was removed, and the pellet was immediately frozen on dry ice and stored at −80 °C until use. Lysates were prepared by resuspension in 60 µl PBS pH 7.8 supplemented with 0.4% (w/v) Triton X-100 and 0.4% (w/v) sodium dodecyl sulfate (SDS), along with protease inhibitor (PI, 1:1000 dilution of protease inhibitor cocktail 3 without EDTA, Calbiochem) and benzonase (Sigma, 1:1,000) at room temperature and incubation at 75 °C for 10 min. Lysates were then cleared by centrifugation for 10 min, 13,000*g* at 15 °C and protein measured by bicinchoninic acid assay (Invitrogen). Click chemistry was performed by adding to 100 μl PBS pH 7.8 (with PI 1:4,000) 20 µl lysate with 100 μg protein, 500 μM triazole ligand Tris[(1-benzyl-1H-1,2,3-triazol-4-yl)methyl]amin (TBTA, Thermo Fisher Scientific, 454531000), 62.5 μM biotin-alkyne tag (Thermo Fisher Scientific, B10185) and 83 μg/ml CuBr (prepared by dilution of a fresh 10 mg/ml solution in dimethyl sulfoxide (DMSO)). The reaction was incubated at room temperature overnight in the dark by overhead rotation. Biotinylated proteins were separated by SDS–PAGE, immunoblotted with anti-GFP and anti-biotin antibodies and IRDye secondary antibodies and scanned with a Licor Odyssey fluorescence imager.

### FUNCAT in cultured neurons

Growth medium was removed and replaced with NGM without methionine (or with methionine in the methionine competition experiments) supplemented with 4 mM ANL or 4 mM methionine as control. Labeling was performed for the specified duration. After metabolic labeling with ANL for the indicated times, cells were washed once fast with medium and were then placed back in medium without ANL for 10 min into the incubator. For protein synthesis inhibition controls, neurons were preincubated for 30 min with 40 µM anisomycin, and 40 µM anisomycin was also present during the incubation with ANL. Neurons were fixed in paraformaldehyde (PFA)-sucrose (PBS pH 7.4 containing 4% sucrose, 4% PFA, 1 mM MgCl_2_ and 0.1 mM CaCl_2_) for 20 min at room temperature, permeabilized with 0.5% Triton in blocking buffer (BB: 4% goat serum in PBS), blocked for 1 h in BB and equilibrated in PBS pH 7.8. The click reaction was performed for 2 h at room temperature using 2 μM of the Alexa647 alkyne (Thermo Fisher Scientific, A10278), in a copper-mediated click reaction with CuSO_4_, Tris(2-carboxyethyl)phosphine (TCEP) and the Triazole ligand TBTA in PBS pH 7.8. Following the click reaction, neurons were washed extensively and blocked with BB for 1 h before immunostaining. Primary antibodies (MAP2 and GFP) were applied for 1 h at room temperature in BB, followed by three washes with PBS (pH 7.4). Secondary antibodies were added for 30 min, and the samples were washed with PBS and water and mounted with AquaPolymount for imaging.

### FUNCAT in acute hippocampal slices

Hippocampal slices (300 µm) were prepared from Nex-Cre::1x/3xMetRS* adult animals. Mice were deeply anesthetized with isoflurane and decapitated. The brain was quickly removed and put in ice-cold, slushed sucrose-based saline solution oxygenated with 95% O_2_/5% CO_2_, cut first along the longitudinal fissure and then cut with an angle of 30° with a vibratome (Leica VT 1200 S) to transverse slices in ice-cold, oxygenated sucrose saline solution. Slices were recovered for 1 h submerged at room temperature in artificial cerebrospinal fluid (ACSF; in mM: NaCl 125, NaHCO_3_ 25, KCl 2.5, NaH_2_PO_4_ 1.25, glucose 10, CaCl_2_ 2 and MgCl_2_ 1) oxygenated with a 95% O_2_/5% CO_2_ gas mixture. Slices were then transferred to an interface chamber, placed on top of lens cleaning paper and continuously perfused with oxygenated ACSF another hour at 32 °C (perfusion rate 2 ml/min). Subsequently slices were labeled for 2 h with or without 1 mM ANL in ACSF by perfusion. Following ANL incubation the slices were perfused for 10 min with ACSF to complete incorporation, rinsed twice in ACSF and fixed in 4% PFA, 4% sucrose in PBS for 1 h at room temperature, then washed in PBS and cryoprotected in 15% and 30% sucrose in PBS overnight. Slices were then resliced to 30 µm with a cryotome and postfixed 10 min with 4% PFA, 4% sucrose in PBS, washed and permeabilized overnight at 4 °C (0.5% Triton X-100 in BB). Samples were washed twice in PBS pH 7.8 and clicked for 3 days. The click chemistry reaction was set up by adding the reagents in the following order: TCEP, TBTA, 2 μM of Alexa647 alkyne, CuSO_4_. After extensive washing, slices were incubated overnight at 4 °C with primary antibodies in BB, washed and incubated for 5 h at room temperature with secondary antibodies in BB. DAPI was added for 5 min in BB. The sections were again washed before mounting on microscope slides. Fluorescence imaging was performed with a LSM780 laser scanning confocal microscope (Zeiss, Zen10 software) using a Plan-Apochromat 20×/numerical aperture (NA) 0.8 M27 objective and appropriate excitation laser lines and spectral detection windows.

### Puromycilation

Neuron cultures from different genotypes were incubated with 3 µM puromycin in growth medium for 5 min at 37 °C, incubation was stopped by two fast washes with prewarmed medium and cells were fixed in PFA-sucrose for 20 min. The cells were then permeabilized for 15 min with 0.5% Triton X-100 in BB, blocked in BB for 1 h and immunostained for puromycin (1 h anti-puromycin, 30 min secondary antibody), then blocked again and poststained for GFP and MAP2 1 h with primary antibodies and 30 min with secondary antibodies and mounted with AquaPolymount for imaging.

### mRNA FISH

For high-sensitivity FISH, cultured neurons were fixed for 20 min with PFA-sucrose. ViewRNA FISH (ViewRNA ISH cell assay, Thermo Fisher Scientific) was performed as described previously^43,44^ using the manufacturer’s detergent for 5-min permeabilization and all probe sets (incubation time 3 h, Thermo Fisher ViewRNA probe sets: *Mars1* type1, cell marker mRNAs type6) and PreAmp/Amp/LP reagents (incubation time 1 h, type1-LP550, type6-LP650) at 1:100 dilution following the manufacturer’s instructions. After mRNA FISH labeling, cells were subjected to MAP2 and GFP immunostaining, mounted in AquaPolymount (Polysciences) and imaged.

For mRNA FISH on hippocampal tissue slices, mice of the respective genotypes were euthanized by decapitation under isoflurane anesthesia; the head was quickly dipped into liquid nitrogen, and the brain was dissected out and fixed in 4% PFA, 4% sucrose in PBS pH 7.4 for 1 h at 4 °C and 1 h at room temperature and cryopreserved in 15% sucrose and 30% sucrose in PBS in overnight steps at 4 °C before cryotome sectioning to 30-µm hippocampal sections. Sections were postfixed for 10 min with the above fixation solution and permeabilized for 20 min with the ViewRNA ISH cell assay detergent solution. The ViewRNA assay was carried out essentially as described above but with overnight incubation in the probe set mixture. Label probes coupled to the 550 dye were used for assays on tissue slices. Subsequent immunostaining was performed by incubating samples with primary antibodies overnight at 4 °C, followed by a 4-h incubation with secondary antibodies. Fluorescence imaging was performed in hippocampal CA1 with a LSM780 laser scanning confocal microscope (Zeiss, Zen10 software) using a Plan-Apochromat 40×/NA 1.4 oil DIC M27 objective and appropriate excitation laser lines and spectral detection windows.

### Cell culture imaging

Eight-bit *Z*-stack images (1,024 × 1,024 pixels) were acquired with a LSM780 confocal microscope (Zeiss) using a Plan-Apochromat 20×/NA 0.8 M27 objective. The detector gain in the signal channel(s) was set to cover the full dynamic range but to avoid saturated pixels. Imaging conditions were maintained identical for each experiment.

### Image analysis and representation

For image quantification, ten randomly chosen regions of each cell culture dish were imaged and maximum intensity projections were used for quantification. For single-cell quantification of FUNCAT, puromycilation or mRNA signal, single somata were manually outlined on the basis of Map2 staining (to distinguish from glial cells), GFP staining or the relevant marker mRNA (FISH), and areas were saved as regions of interest (ROIs) in ImageJ. Then, images were split into single channels and the mean intensity value for each ROI was measured in the relevant signal channels. Splitting the neuron population into Cre-negative and Cre-positive neurons within a dish was based on fixed thresholds for FUNCAT signal or GFP signal set by comparison with no-ANL- and/or Cre-negative culture controls. In the protein synthesis inhibitor experiments, FUNCAT mean gray values were calculated for each condition from whole images in the signal channel (ten randomly chosen regions per dish). ROI or whole-image quantifications were exported from ImageJ, calculations were performed in Microsoft Excel and graphs were plotted in GraphPadPrism. Statistical analysis was performed in GraphPadPrism using ANOVA and correction for multiple comparisons.

For quantification of the *Mars1* signal in tissue sections, maximum intensity projections of the images from different genotypes were used from three sets of mice. Two 100 × 200 µm boxes were placed in each image in a way that the border from stratum pyramidale to stratum radiatum was aligned between the samples, and a line profile was calculated in ImageJ for each box along the longer axis. Profiles of the same genotype were averaged along the stratum pyramidale–stratum radiatum axis in GraphPad Prism. To estimate the difference in *mars1* signal between Nex-Cre::3xMetRS* and Nex-Cre::1xMetRS* and wild-type MetRS, we calculated the area under the averaged profile curves for each genotype in GraphPadPrism using the last value as baseline and subtracted the calculated area from the 3xMetRS* and 1xMetRS* without Cre, respectively. For image representations, maximum intensity projections were split into the single channels. In some cases, the channel to be displayed was converted to inverted grayscale images or fire lookup table (LUT) for better visualization, and brightness and contrast was enhanced in the same way for all displayed images within one experiment in ImageJ.

### ANL labeling and administration to living animals

For in vivo labeling, 8- to 10-week-old Cre::floxed-3xMetRS* or age-matched controls (referred as no-ANL control in the text) mice were labeled by a single IP injection with 300 mM ANL (pH 7.5; 1:10 µl of body weight) and euthanized at the specified times; both male and female mice were used. Animals were maintained as double homozygous (Nex-Cre::3xMetRS*, DAT-Cre::3xMetRS* and Nex-Cre::1xMetRS*); for 3× and 6× comparison experiments, double heterozygotes were obtained by mating them with wild-type animals. At the end of the labeling period, mice were euthanized with CO_2_ (following local lawfare). For FUNCAT experiments, animals were perfused with 4% PFA, treated with sucrose and cut at 30 µm. After click chemistry (performed as described above), fluorescence images were acquired using a microscope (DM 1000, Leica). Sections were photographed with Plan 4× dry objective lens (NA 0.1) at room temperature. For BONCAT experiments, brain tissue was removed, dissected and frozen in dry ice.

### BONCAT in tissue

Dissected tissue was homogenized and lysed in PBS supplemented with 1% (w/v) Triton X-100 and 1% (w/v) SDS, along with PI (1:750 dilution of protease inhibitor cocktail 3 without EDTA, Calbiochem) and benzonase (Sigma, 1:1,000) at 75 °C for 15 min. Lysates were then cleared by centrifugation and stored at −80 °C until use. BONCAT was performed as previously described^45^. In brief, click chemistry was performed in 120 μl PBS (pH 7.8) supplemented with 0.08% Triton (w/v) SDS, 0.2% (w/v), 90 μg proteins, 300 μM triazole (Sigma, ref. 678937), 50 μM biotin-alkyne tag (Thermo, ref. B10185) or DST (disulfide tag alkyne) (Click Chemistry Tools) and 83 μg/ml CuBr (prepared by dilution of a fresh 10 mg/ml solution in DMSO). The reaction was incubated at 4 °C overnight in the dark. Biotinylated proteins were then separated by electrophoresis and immunoblotted with anti-GFP and anti-biotin antibodies. Tissue samples were lysed as explained before; cleared extracts were alkylated twice with 20 mM iodoacetamide (3 h at room temperature in the dark) and then cleaned by two passages on PD-SpinTrap G-25 columns (GE Healthcare). Samples were then clicked as explained before. After click chemistry, excess biotin-alkyne was removed by protein precipitation with trichloroacetic acid (TCA). Tagged proteins were then affinity-purified with high-capacity Neutravidin agarose beads (Thermo) in PBS supplemented with 1% Triton, 0.15% SDS and PI (binding buffer) overnight at 4 °C. The beads were then washed extensively at room temperature, first in binding buffer, then in 0.4% SDS and finally with PBS + PI and 50 mM ammonium bicarbonate + PI. Bound proteins were eluted by incubation with 5% β-mercaptoethanol, 0.03% SDS and PI for 30 min at room temperature. Two consecutive elutions were performed. Each replicate in the BONCAT for western blots and for MS at 3-h and 6-h time points is a single animal; for the OB MS, two animals were used.

### Sample processing for liquid chromatography (LC)–MS/MS

Eluted proteins were prepared for bottom-up proteomics using a suspension trapping protocol as previously reported^46^. In brief, samples were mixed with lysis buffer (10% SDS, 100 mM Tris, pH 7.55, with H_3_PO_4_) in a 1:1 ratio, reduced using 20 mM dithiothreitol for 10 min at room temperature and alkylated with 50 mM iodoacetamide for 30 min at room temperature in the dark. Subsequently, samples were acidified using phosphoric acid to a final concentration of 1.2%. Then, binding/wash buffer (90% methanol, 50 mM Tris, pH 7.1 with H_3_PO_4_) was added in a 1:7 lysate-to-buffer ratio. The protein suspension was loaded onto S-trap filters (size: ‘micro’; ProtiFi) by centrifugation for 20 s at 4,000*g*. Trapped proteins were washed with 150 μl of binding/wash buffer buffer four times. Trypsin (1 μg; Promega) was added in 60 μl of 40 mM ammonium bicarbonate buffer. Digestion was performed overnight (~18 h) at room temperature in a humidified chamber. Peptides were collected by washing through three consecutive steps by centrifugation at 4,000*g* for 40 s, starting with the digestion buffer and followed by two washes with 0.2% formic acid, in MS-grade water. Peptides were dried in vacuo at 45 °C.

### LC–MS/MS analysis

Dried peptides were reconstituted in 95% MS-grade H_2_O, 5% acetonitrile (ACN) and 0.1% trifluoroacetic acid (TFA). For the time course experiment (3 h and 6 h ANL labeling), peptides were loaded onto a C18-PepMap 100 trapping column (particle size 3 µm, length *L* = 20 mm; Thermo Fisher Scientific) and separated on a C18 analytical column with an integrated emitter (particle size 1.7 µm, inner diameter 75 µm, *L* = 50 cm; CoAnn Technologies) using a nano-HPLC (Dionex U3000 RSLCnano) coupled to a nanoFlex source (2000 V, Thermo Fisher Scientific). The temperature of the column oven (SonationAnalytics) was maintained at 55 °C. Trapping was carried out for 6 min with a flow rate of 6 μl/min using a loading buffer (100% H_2_O and 2% ACN with 0.05% trifluoroacetic acid). Peptides were separated by a gradient of water (buffer A: 100% H_2_O and 0.1% FA) and ACN (buffer B: 80% ACN, 20% H_2_O and 0.1% FA) with a constant flow rate of 250 nl/min. In 155 min runs, peptides were eluted by a nonlinear gradient with 120-min active gradient time, as selected and reported for the respective MS method by Muntel et al.^47^. Analysis was carried out on a Fusion Lumos mass spectrometer (Thermo Fisher Scientific) operated in positive polarity and data-independent acquisition^48^ mode. In brief, the 40-window DIA method had the following settings: full scan: orbitrap resolution 120k, automatic gain control (AGC) target 125%, mass range 350–1,650 *m*/*z* and maximum injection time 100 ms. DIA scan: activation type: higher‑energy collisional dissociation (HCD), HCD collision energy 27%, orbitrap resolution 30k, AGC target 2,000% and maximum injection time dynamic. For the cell type-specific analysis of the OB, peptides were analyzed using a nanoElute 1 nano-HPLC coupled to a timsTOF Pro II mass spectrometer via a captive spray ion source (1,600 V, Bruker Daltonics). Peptides were loaded directly onto the analytical column (15 cm × 75 µm column with 1.9-μm C18-beads (PepSep); at a maximum of 800 bar), maintained at 60 °C and connected to a 20-µm zero‑dead‑volume (ZDV) sprayer (Bruker Daltonics). In 37-min runs, peptides were separated in a linear gradient of water (buffer A: 100% H_2_O and 0.1% FA) and ACN (buffer B: 100% ACN and 0.1% FA), ramping from 2% to 35% in 30 min with a constant flow rate of 500 nl/min. For data acquisition in DIA mode, the ‘short-gradient’ DIA-PASEF (parallel accumulation–serial fragmentation) method was used. In brief, 21 DIA-PASEF windows were distributed to a trapped ion mobility spectrometry (TIMS) scan each and designed to cover an *m*/*z* range from 475 to 1,000 *m*/*z* in 25-Da windows, leading to an estimated method cycle time of 0.95 s. The ion mobility range was set from 1.30 to 0.85 Vs/cm^2^. Further details on the method parameters are embedded in the uploaded raw data.

### Data analysis of DIA LC–MS/MS data

DIA raw files were processed with the open-source software DIA-NN (version 1.8.2 beta 27) using a library-free approach. The predicted library was generated using the in silico FASTA digest (Trypsin/P) option with the UniProtKB database (Proteome_ID: UP000000589) for *Mus musculus*. Deep learning-based spectra and retention time prediction were enabled. The covered peptide length range was set to 7–35 amino acids, missed cleavages to 2 and precursor charge range to 1–5. N-terminal methionine excision, methionine oxidation and N-terminal acetylation were set as variable modifications, with cysteine carbamidomethylation as a fixed modification. The maximum number of variable modifications per peptide was limited to 3. According to most of DIA-NN’s default settings, MS1 and MS2 mass accuracies, as well as scan windows, were set to 0; isotopologs and match-between-runs were enabled, while shared spectra were disabled. Protein inference was performed using genes with the heuristic protein inference option enabled. The neural network classifier was set to single-pass mode, and the quantification strategy was selected as ‘QuantUMS (high precision)’. The cross-run normalization was set to ‘RT-dependent’, the library generation to ‘smart profiling’ and the speed and RAM usage to ‘optimal results’. No normalization was applied (additional option ‘--no-norm’). The DIA-NN report table and the respective FASTA file were imported into the statistical computing software R and analyzed using the MSDAP R package^49^. MSDAP performed protein inference and label-free quantification at the protein level (minimum of one peptide per protein).

### Data analysis of cell type-specific proteomics

To assess cell type-specific enrichment, ANL-labeled samples were compared with a no-ANL control. First, a comparison was performed based on valid protein quantifications in both conditions (‘exclusivity approach’), and second, relative enrichment over control was examined (‘enrichment approach’). For differential abundance analysis, a peptide-centric linear mixed-effect model as implemented in the msqRob algorithm in the MSDAP pipeline was used. Multiple testing correction was performed according to the Benjamini–Hochberg method. For the DA proteome, proteins were counted as exclusively quantified in the labeled samples (and thus part of the DA proteome) if four of seven DA samples and none of the negative control samples had valid protein quantifications. Significantly enriched proteins had to be quantified in at least four of seven labeled and one of five control samples, have a *q* value <0.01 and show at least 1.5-fold enrichment over control (log_2_ fold change ~0.58). For the excitatory proteome, proteins were exclusively quantified in the labeled samples (and thus part of the excitatory proteome) if five of six excitatory samples and none in the negative control samples had valid values. Significantly enriched proteins had to be quantified in at least five of six labeled and one of two control samples, have a *q* value <0.01 and show at least threefold enrichment over the control (log_2_ fold change ~1.58). To assess changes in protein intensity after prolonged labeling duration, the DIA-NN precursor report was filtered for proteins matching the intersection of the previously identified excitatory proteome of the two time points, before performing the linear mixed-effect model in MSDAP (*q* value <0.01, 1.5-fold enrichment). To compare the DA and excitatory neuronal proteomes with previously published studies, a gene-level approach was used to integrate the different datasets. Before merging, any information on protein isoforms (for example, protein half-life) was aggregated to gene IDs by their mean value. Gene Ontology and PANTHER pathway overrepresentation analyses were performed using ShinyGO v0.80^50^ with a custom background list of the overall OB proteome derived from Sharma et al.^25^, applying an FDR cutoff of 0.05 and plotting in order of lowest FDR (top terms). All visualizations were performed using base R or the ggplot2 package.

### Reporting summary

Further information on research design is available in the Nature Portfolio Reporting Summary linked to this article.

## Online content

Any methods, additional references, Nature Portfolio reporting summaries, source data, extended data, supplementary information, acknowledgements, peer review information; details of author contributions and competing interests; and statements of data and code availability are available at 10.1038/s41684-025-01589-2.

## Supplementary information

Supplementary Information Supplementary figures.

Reporting Summary

Supplementary Data All the identified protein candidates by MS.

## Source data

Source Data Fig. 2 Unprocessed western blot.

Source Data Fig. 4 Unprocessed western blots.

Source Data Fig. 5 Unprocessed western blots.

## Data Availability

All MS proteomics raw data and associated search engine results have been deposited to the ProteomeXchange Consortium via the PRIDE partner repository^51^ with the dataset identifier PXD057536. Analysis scripts and any other data are available upon request. Source data are provided with this paper.
