# Supplementary material for: Cell type-specific in vivo proteomes with a multicopy mutant methionyl tRNA synthetase mouse line
Source: Lab Anim (NY). 2025 Aug 13;54(9):228–37. doi: 10.1038/s41684-025-01589-2 (PMC12404990; doi:10.1038/s41684-025-01589-2)
Supplement: Supplementary file 1 — Supplementary figures. [file 41684_2025_1589_MOESM1_ESM.pdf]

lab animal

Supplementary information

https://doi.org/10.1038/s41684-025-01589-2

Cell type-specific in vivo proteomes with a multicopy mutant methionyl tRNA synthetase mouse line

In the format provided by the authors and unedited

Supplementary Figure 1

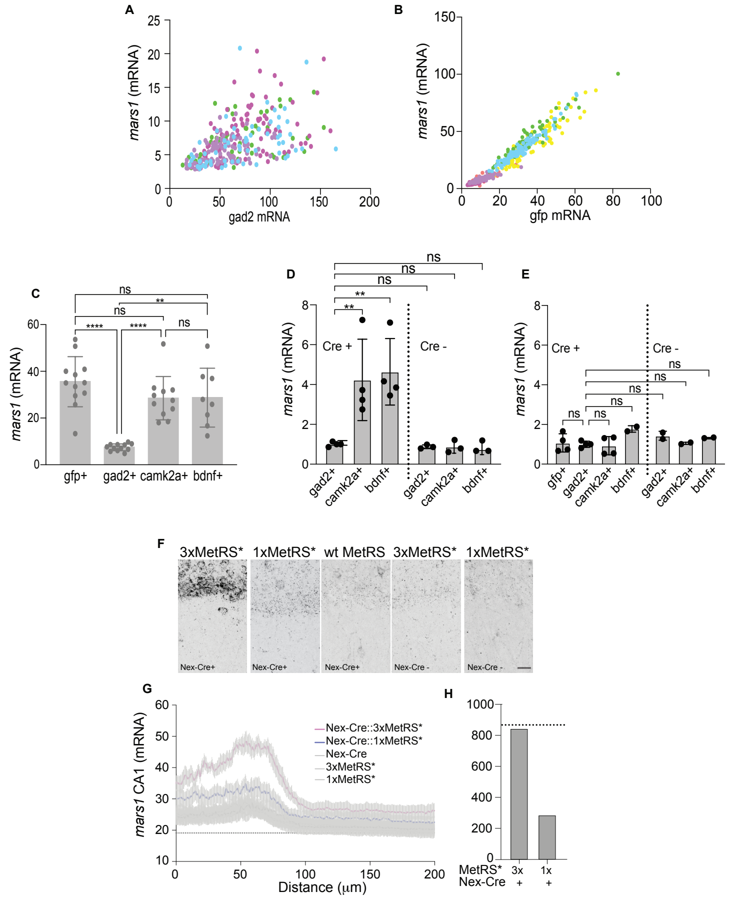

Supplementary Figure 1. FISH for MetRS mRNA (*mars1)* in cultured neurons and hippocampal slices. (A), FISH signals for *mars1* and *gad2* (mRNA) are distributed in the same intensity space in inhibitory cells (*gad2*+) from Nex-Cre::3xMetRS* (blue, green dots) and Nex-Cre::1xMetRS* (pink, purple dots) cultures. Data points shown are independent dishes, total number of cells/number of dishes, (**B),** Both, mRNA FISH signals for *gfp* and *mars1* are substantially higher in *gfp*+ cells from Nex-Cre::3xMetRS* (blue, green, yellows) than from Nex-Cre::1xMetRS* (pink, purple, reds) cultures. Data points shown are independent dishes, total number of cells/number of dishes, (**C),** Quantification of *mars1* in different cell types identified by FISH signal (*gfp+, gad2*+, *camk2a*+ or *bdnf*+,) within cultured neurons from Nex-Cre::3xMetRS* mice. Excitatory cells (*camk2a+, bdnf*+) have comparable *mars1* signal to *gfp*+ neurons while inhibitory cells (*gad2*+) have lower *mars1* signal. (Multiple comparison ANOVA p<0.0001, ** p=0.009). Data points shown are independent dishes, total number of cells/number of dishes, gfp+ (726/12), gad2+ (640/12), camk2a+ (695/11), bdnf+ (369/8)). (**D, E)** Quantification of *mars1* in different cell types identified by FISH signal (*gad2*+, *camk2a*+ or *bdnf*+) in primary cultured neurons from 3xMetRS* (Multiple comparison ANOVA ** p=0.0080 (gad2+/camk2a+), ** p=0.0030 (gad2+/bdnf+)), **(D)** or 1xMetRS*. Data points shown are independent dishes, total number of cells/number of dishes, CRE+: gad2+ (261/4), camk2a+ (269/4), bdnf+ (199/4) CRE -: gad2+ (133/3), camk2a+ (201/3), bdnf+ (151/3). **(E)** Mice expressing Nex-Cre (left part) or not (right part) show significant overexpression of *mars1* only in Nex-Cre::3xMetRS* excitatory cells. The signal is normalized on *mars1* in *gad2*+ neurons from Nex-Cre-positive cultures. **(F)**, Confocal images of FISH detecting MetRS mRNA in the CA1 layer of the hippocampus in brain slices from F1 mice expressing Nex-Cre and 1x/3xMetRS* (left part) and their homozygous parent lines Nex-Cre (only wt MetRS, middle panel) or homozygous 1x/3x MetRS* (without Nex-Cre, right part). Scale bar 25 μm. (**G)**, Quantification of MetRS mRNA as a function of the distance to the CA1 pyramidal layer shown in F,

average fluorescence of 10 areas/genotype were quantified, error bars represent SEM. (**H)**, Quantification of the area under the curve for MetRS* mRNA expression shown in F.

Supplementary Figure 2

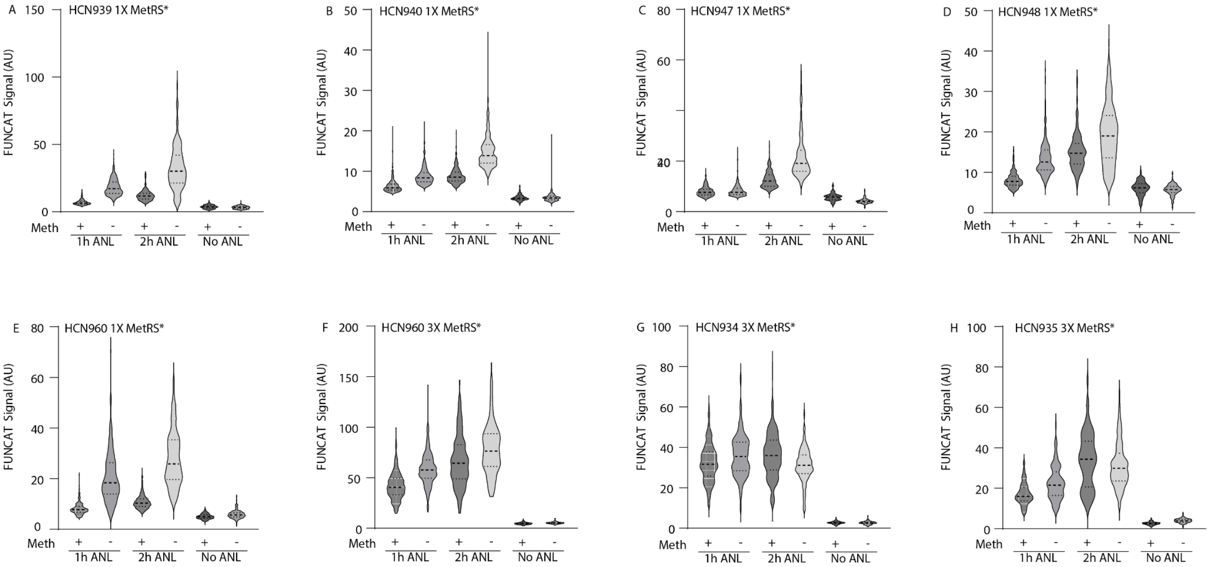

Supplementary Figure 2. Methionine competition with ANL incorporation to MetRS*. Violin plots representing the FUNCAT quantification of each neuron from the primary neuronal cultures shown in Fig. 3, from Nex-Cre::1xMetRS* and Nex-Cre::3xMetRS* mice, labeled with ANL in culture media with (+) or without (-) methionine for 1 hour and 2 hours. In each graph (A-H), a single experiment is represented.

Supplementary Figure 3

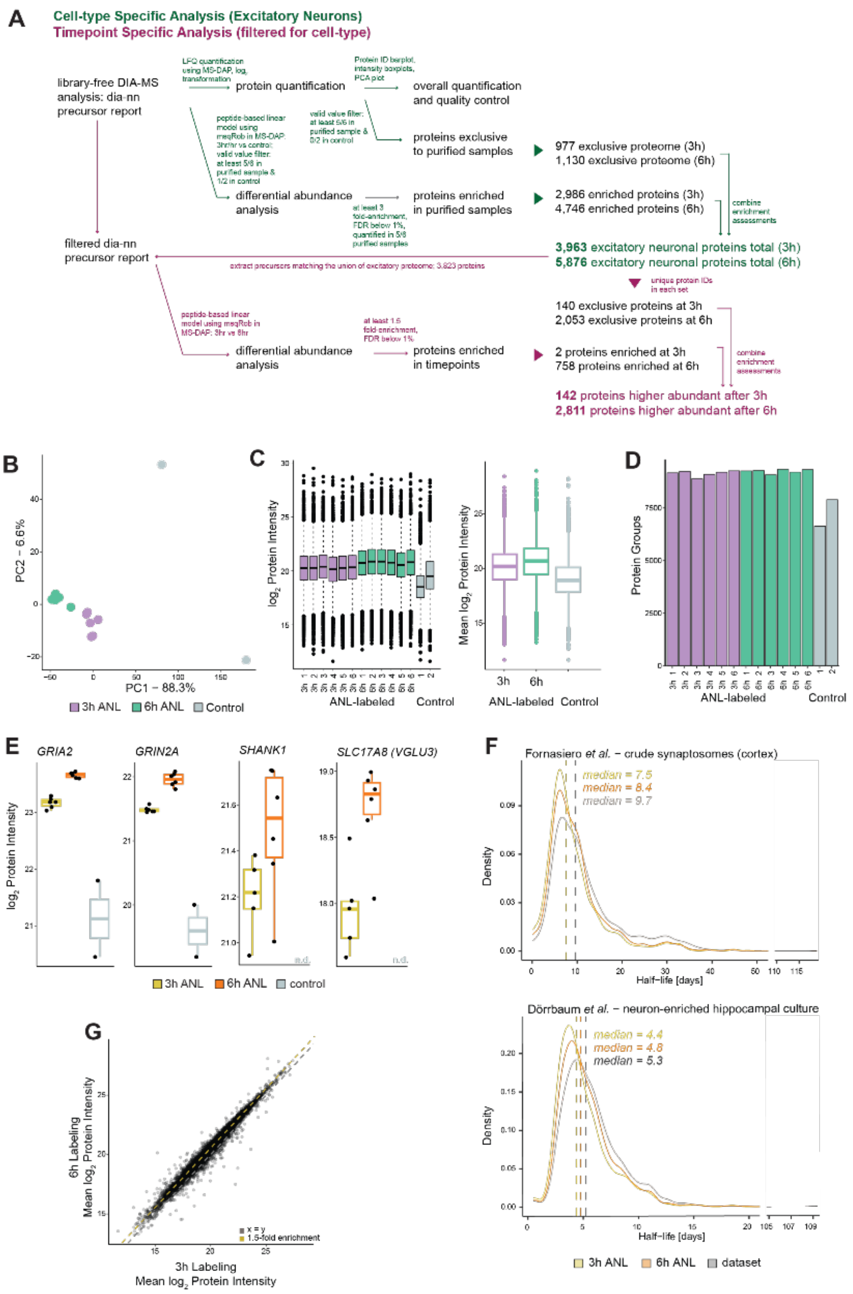

Supplementary Figure 3. Proteomic analysis of excitatory neuronal proteins from mouse cortex using short labeling *in vivo*. (**A**) Detailed bioinformatic analysis pipeline for the characterization of the excitatory neuronal proteome from the cortex after 3 hours and 6 hours of labeling with ANL *in vivo*. All relevant steps of the two-staged analysis—cell type specificity [2] and temporal specificity (maroon)—are indicated. (**B**) Principal component analysis (PCA) of overall protein group quantifications (log2) showing clustering of biological replicates and separation of conditions, indicating similarity between the proteins after 3 hours and 6 hours of labeling (>88% variance explained by component 1). (**C**) Log2-

scaled protein intensities of the identified proteins for each biological replicate (left) and log2 mean intensities for each time point and control samples (right). (**D**) Box plots of overall protein group intensities (log2) for each condition, separated by biological replicates (left) or merged by mean values (right). (**E**) Box plots display selected excitatory candidate proteins showing either significant enrichment or exclusive detection in the labeled conditions. (**F**) The density plot shows protein half-life distribution in mouse cortical synaptosomes (left) or neuron-enriched hippocampal cultures (right)[19]. Excitatory neuronal proteins identified at each time point are matched to previously published databases at the level of gene symbols and are highlighted by color; overall half-life data is depicted in gray. Vertical lines highlight lower median half-lives of the matched excitatory proteins after 3 hours than after 6 hours, both having shorter half-lives compared to the full proteome. (**G**) Scatter plot of mean log2 of protein intensities of all identified proteins at 3 hours and 6 hours, showing a shift towards higher intensities at 6 hours. See also panel C (right). Boxes indicate the median, first, and third quartiles, and whiskers extend to 1.5 × IQR.

Supplementary Figure 4

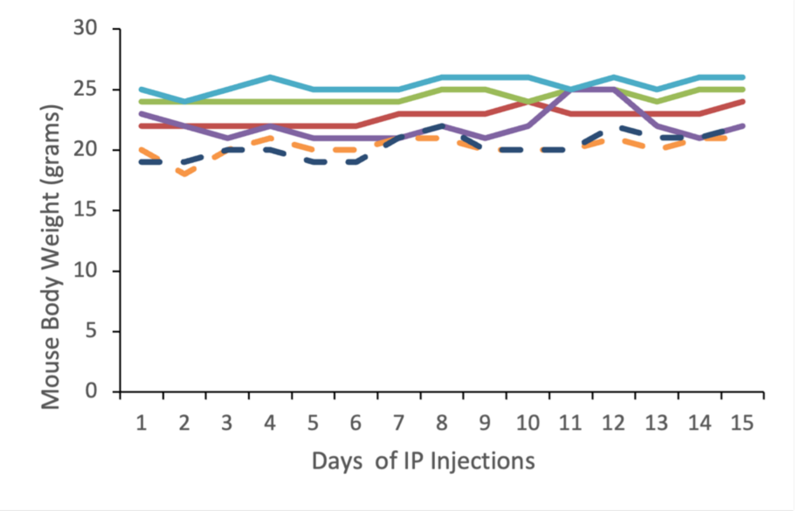

Supplementary Figure 4. Mouse body weight during the 15 day injection protocol. Mouse weights were monitored daily over the complete course of IP injections. No significant weight loss was observed, indicating a good tolerance to ANL.

Supplementary Figure 5

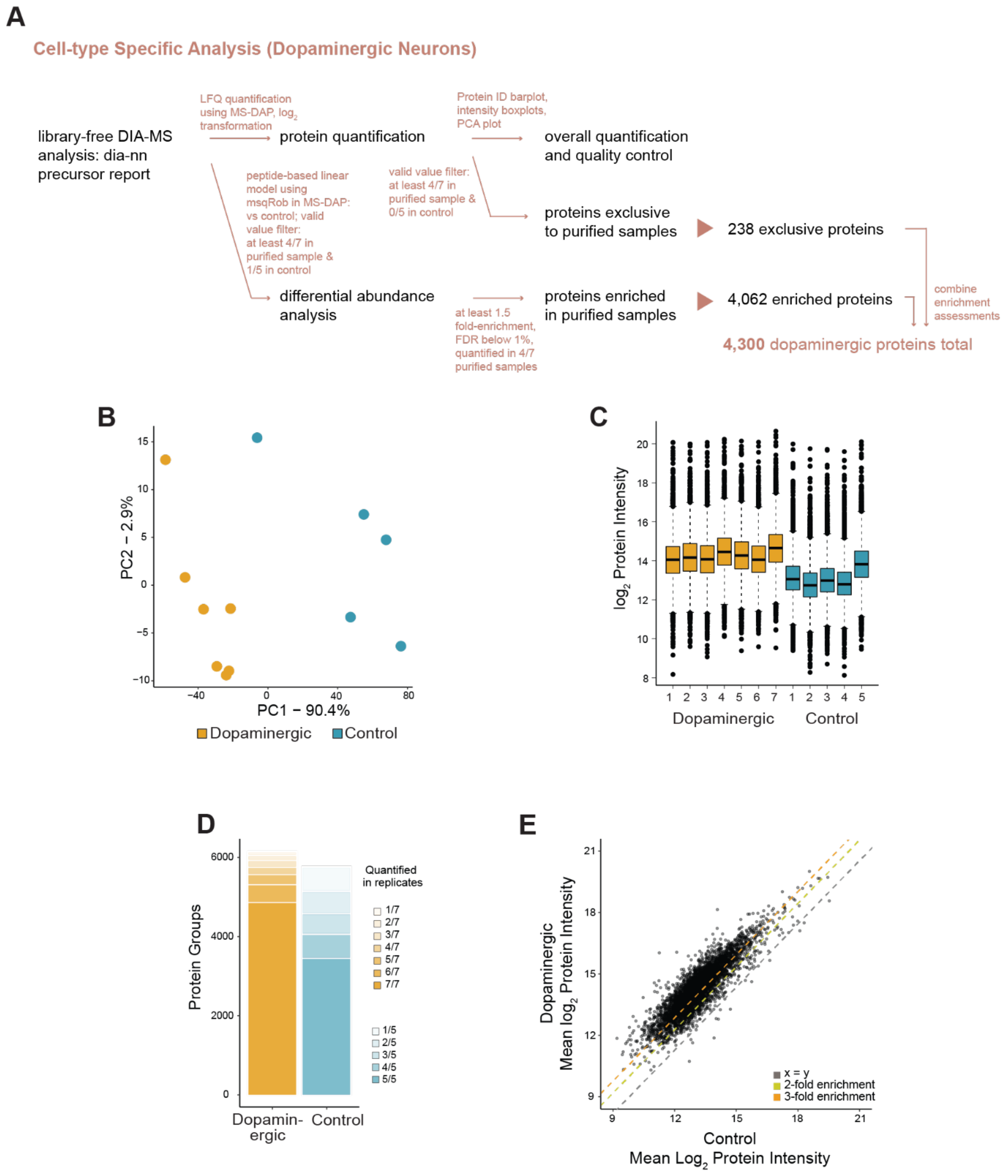

Supplementary Figure 5. Proteomic analysis of the dopaminergic neuronal proteome of the olfactory bulb using *in vivo* labeling. (A) Detailed bioinformatic analysis pipeline for the characterization of the OB-DA proteome after two weeks of labeling by daily IP injections of ANL. All relevant steps of the cell type-specific analysis are indicated. (**B**) Principal component analysis of overall protein group quantifications (log2) showing separation of the labeled vs. unlabeled samples (<90.4% variance explained by component 1) and clustering of biological replicates. (**C**) Boxplots of overall protein group intensities (log2) for each condition, separated by biological replicates. Boxes specify the median, first and third quantile, and whiskers extend to 1.5 × IQR. (**D**) Bar plot of overall protein quantifications for the labeled dopaminergic and unlabeled control samples. Saturation of the stacked bars indicates the number of valid observations for a protein group in all biological replicates. (**E**) Scatterplot of mean log2 protein group intensities of all identified proteins in the unlabeled negative control vs. labeled samples, showing a shift toward higher intensities for the labeled dopaminergic proteins. See also panel C.
