# Supplementary figures and images for: Cell type-specific in vivo proteomes with a multicopy mutant methionyl tRNA synthetase mouse line
Source: Lab Anim (NY). 2025 Aug 13;54(9):228–37. doi: 10.1038/s41684-025-01589-2 (PMC12404990; doi:10.1038/s41684-025-01589-2)

## Slide 1
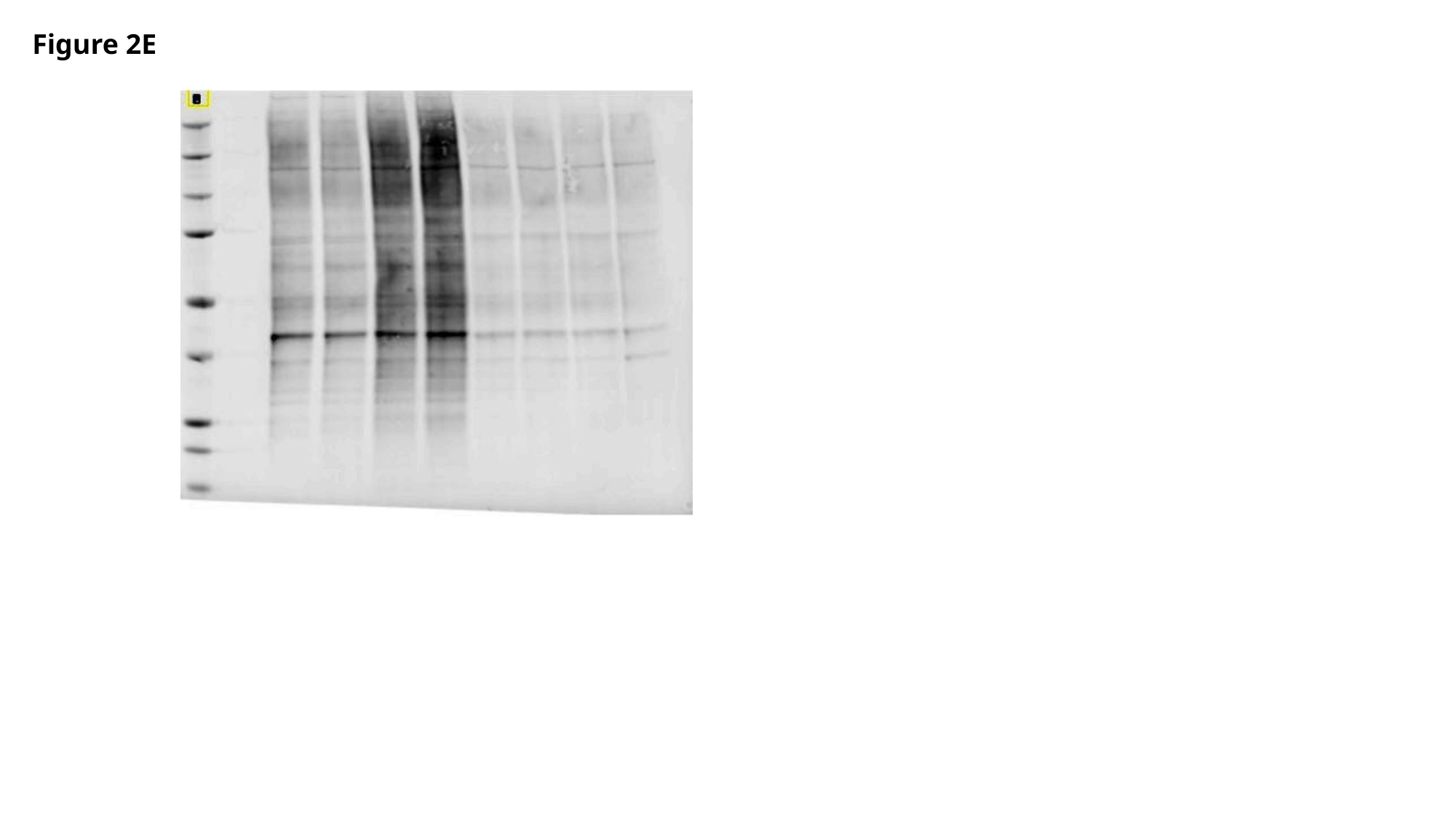

Figure 2E

Supplement: Supplementary file 4 — Unprocessed western blot. [file 41684_2025_1589_MOESM4_ESM.pptx]

## Slide 1
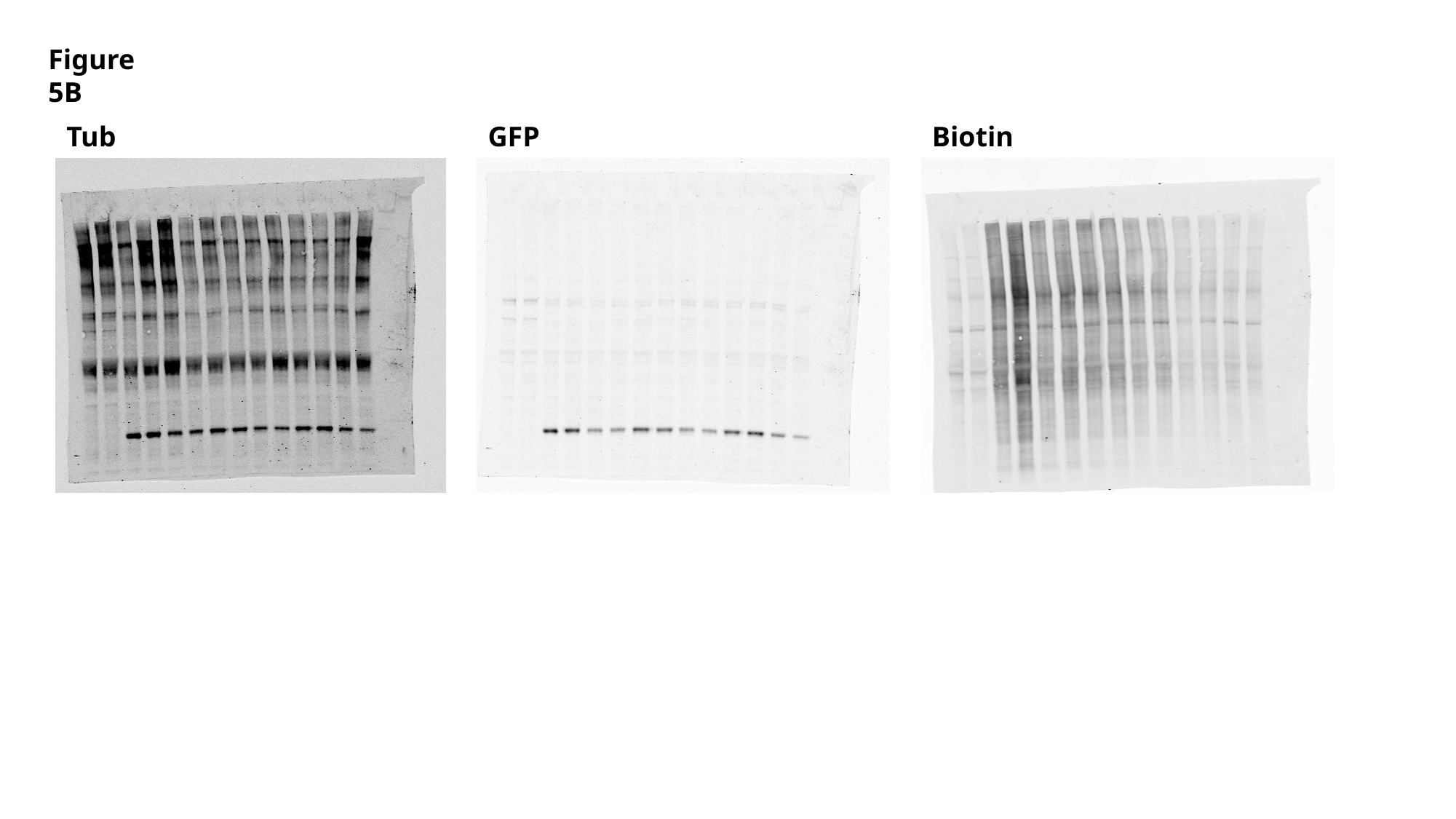

Figure 5B
GFP
Tub
Biotin

Supplement: Supplementary file 6 — Unprocessed western blots. [file 41684_2025_1589_MOESM6_ESM.pptx]
