# Supplementary material for: Cell type-specific in vivo proteomes with a multicopy mutant methionyl tRNA synthetase mouse line
Source: Lab Anim (NY). 2025 Aug 13;54(9):228–37. doi: 10.1038/s41684-025-01589-2 (PMC12404990; doi:10.1038/s41684-025-01589-2)
Supplement: Supplementary file 5 — Unprocessed western blots. [file 41684_2025_1589_MOESM5_ESM.pptx]

## Slide 1
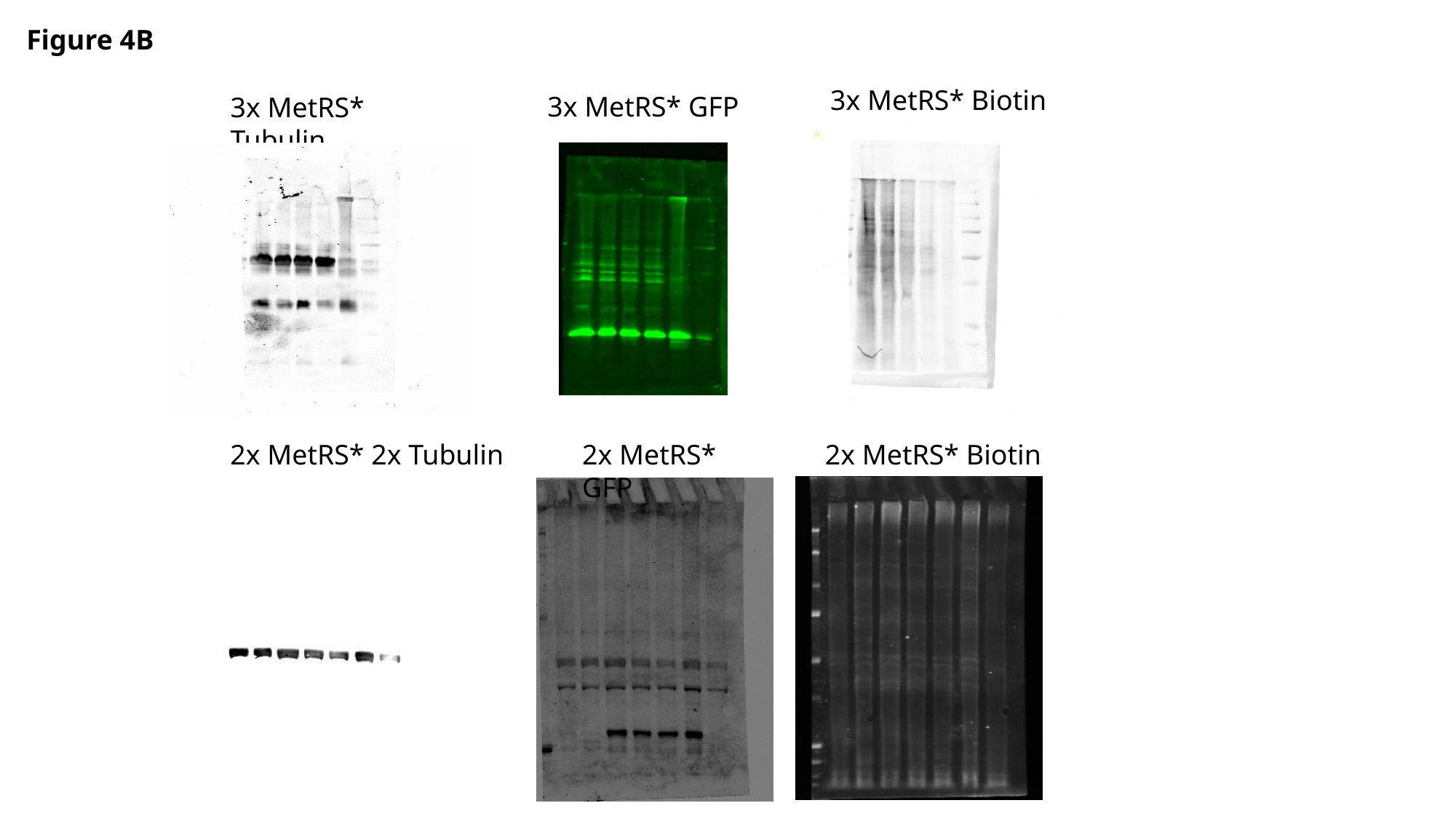

Figure 4B
3x MetRS* Biotin
3x MetRS* GFP
3x MetRS* Tubulin
2x MetRS* 2x Tubulin
2x MetRS* GFP
2x MetRS* Biotin
